# Unravelling the ontogeny of a Devonian early gnathostome, the “acanthodian” *Triazeugacanthus affinis* (eastern Canada)

**DOI:** 10.7717/peerj.3969

**Published:** 2017-10-27

**Authors:** Marion Chevrinais, Jean-Yves Sire, Richard Cloutier

**Affiliations:** 1 Laboratoire de Paléontologie et Biologie évolutive, Université du Québec à Rimouski, Rimouski, Canada; 2 CNRS—UMR 7138-Evolution Paris-Seine IBPS, Université Pierre et Marie Curie, Paris, France

**Keywords:** Gnathostomata, Acanthodii, Mineralization, Developmental trajectory, Ossification sequence

## Abstract

The study of vertebrate ontogenies has the potential to inform us of shared developmental patterns and processes among organisms. However, fossilised ontogenies of early vertebrates are extremely rare during the Palaeozoic Era. A growth series of the Late Devonian “acanthodian” *Triazeugacanthus affinis*, from the Miguasha *Fossil*-*Fish Lagerstätte*, is identified as one of the best known early vertebrate fossilised ontogenies given the exceptional preservation, the large size range, and the abundance of specimens. Morphological, morphometric, histological and chemical data are gathered on a growth series of *Triazeugacanthus* ranging from 4 to 52 mm in total length. The developmental trajectory of this Devonian “acanthodian” is characteristic of fishes showing a direct development with alternating steps and thresholds. Larvae show no squamation but a progressive appearance of cartilaginous neurocranial and vertebral elements, and appendicular elements, whereas juveniles progress in terms of ossification and squamation. The presence of cartilaginous and bony tissues, discriminated on histological and chemical signatures, shows a progressive mineralisation of neurocranial and vertebral elements. Comparison among different body proportions for larvae, juveniles and adults suggest allometric growth in juveniles. Because of the phylogenetic position of “acanthodians”, *Triazeugacanthus* ontogeny informs us about deep time developmental conditions in gnathostomes.

## Introduction

**Figure 1:**
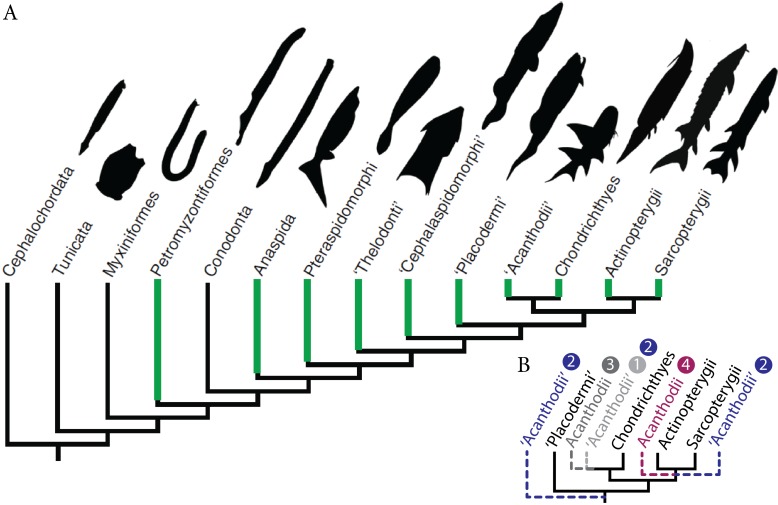
Fossil ontogenies in vertebrates. (A) Taxa with fossil ontogenies in green. Ontogenetic data from Cloutier (2010). Figure modified from Larouche, Zelditch & Cloutier (2017). (B) Alternative hypotheses of “acanthodians” phylogenetic position, numbers refer to hypotheses proposed in the text.

Historically, the identification of vertebrate fossilised ontogenies has often been overlooked because distinct morphologies have been frequently assigned to different species rather than different ontogenetic stages of the same species (Cloutier et al., 2009; Donoghue & Purnell, 2009; Horner & Goodwin, 2009; Cloutier, 2010; Delfino & Sánchez-Villagra, 2010; Sánchez-Villagra, 2010). Although the recognition of developmental stages (i.e., embryonic, larval, juvenile, adult and senescent) is difficult, Palaeozoic fossilised ontogenies have been recorded in most major clades of early vertebrates from basal jawless fish to advanced pre-tetrapod sarcopterygians (Cloutier, 2010) (Fig. 1A). Descriptions of fossilised ontogenies necessitate the recognition of key patterns and processes, even when organisms are weakly mineralised during early stages of life; this requires exceptional preservation.

Palaeozoic early gnathostomes are represented by four major groups, namely the “placoderms” (Early Silurian to Late Devonian), “acanthodians” (Late Ordovician to Middle-Late Permian), chondrichthyans (Late Ordovician to Recent) and osteichthyans (Late Silurian to Recent). The phylogenetic position and status of both “placoderms” and “acanthodians” is still a matter of debate (Brazeau, 2009; Davis, Finarelli & Coates, 2012; Zhu et al., 2013; Brazeau & Friedman, 2015; Burrow et al., 2016; Qu et al., 2016; Chevrinais, Sire & Cloutier, 2017). The “placoderms” are either considered as a paraphyletic group at the base of other gnathostomes (Young, 2010; Zhu et al., 2013; Dupret et al., 2014; Long et al., 2015) or a monophyletic sister-group of either chondrichthyans or osteichthyans (Young, 2010). “Acanthodians” are either considered (1) stem chondrichthyans (Zhu et al., 2013; Long et al., 2015; Burrow et al., 2016; King et al., 2017; Chevrinais, Sire & Cloutier, 2017) or (2) stem gnathostomes, stem chondrichthyans, and stem osteichthyans (Brazeau, 2009; Davis, Finarelli & Coates, 2012), (3) the monophyletic sister-group to chondrichthyans (Dupret et al., 2014) or (4) osteichthyans (Schultze, 1990; Hanke & Davis, 2012) (Fig. 1B). Ontogenetic data on “placoderms” and “acanthodians”, provided by the description and understanding of their early stages of development, are of paramount importance for resolving the early vertebrate phylogeny because developmental data represent an underused source of phylogenetic data.

For more than 30 years, “acanthodian” growth series have been recognised but frequently based on limited size series including already large individuals (Chevrinais, Sire & Cloutier, 2017, see review). Nevertheless, more than 15 ontogenies have been documented: one possible Ischnacanthiformes (*Nerepisacanthus denisoni* (Burrow & Rudkin, 2014)), two Diplacanthiformes (*Diplacanthus horridus* (Cloutier et al., 2009), *Uraniacanthus curtus* (Newman et al., 2012)), one Climatiiformes (*Tetanopsyrus breviacanthias* (Hanke, Davis & Wilson, 2001)), two species of uncertain order (*Machaeracanthus goujeti* (Botella, Martinez-Perez & Soler-Gijon, 2012), *Lupopsyrus pygmaeus* (Hanke & Davis, 2012)), and nine Acanthodiformes (*Lodeacanthus gaujicus* (Upeniece, 1996; Upeniece, 2001; Upeniece & Beznosov, 2002), *Triazeugacanthus affinis* (Chevrinais, Cloutier & Sire, 2015; Chevrinais, Balan & Cloutier, 2015; Chevrinais, Sire & Cloutier, 2017), *Homalacanthus concinnus* (Cloutier et al., 2009), *Acanthodes bridgei* (Zidek, 1985), *A. bronni* (Heidtke, 1990), *A. gracilis* (Zajic, 2005), *A. lopatini* (Beznosov, 2009), *A. ovensi* (Forey & Young, 1985), and an acanthodiform indet. (Coates, 1993)).

Three “acanthodian” growth series (i.e., *Diplacanthus horridus*, *Triazeugacanthus affinis*, and *Homalacanthus concinnus*) have been described from the middle Frasnian (*ca*. 380 Ma) Escuminac Formation (Miguasha, Quebec, Canada) which yielded fossilised ontogenies for 14 out of the 20 Escuminac vertebrate species (Cloutier et al., 2009). Recently, the ontogeny of *Triazeugacanthus* has been reinvestigated (Chevrinais, Balan & Cloutier, 2015; Chevrinais, Cloutier & Sire, 2015; Chevrinais, Sire & Cloutier, 2017) showing significant increases with total length in (1) the size of individual anatomical elements, (2) the number of skeletal elements, and (3) the squamation extent, as well as (4) the progressive mineralisation of skeletal elements with growth.

Our aims are (1) to describe the ontogeny of *Triazeugacanthus* in terms of sequence of ossification and morphometric changes and (2) to compare the developmental sequence and trajectory of *Triazeugacanthus* to that reported in other “acanthodians”, chondrichthyans, and osteichthyans. Because of the hypothesized stem chondrichthyan phylogenetic position of “acanthodians” (Chevrinais, Sire & Cloutier, 2017), we expect *Triazeugacanthus* to be informative on patterns of development shared by a large array of gnathostomes.

## Material and Methods

### Material

Specimens of *Triazeugacanthus affinis* (MHNM and NMS collections) were observed under water immersion (Leica MZ9.5), drawn using a camera lucida, and photographed (Nikon D300, Tokyo, Japan). *Triazeugacanthus* ontogenetic stages were recognised originally based on distinctive characteristics (Cloutier, 2010; Chevrinais, Cloutier & Sire, 2015): (1) larvae are identified by the absence of body scales (Urho, 2002; Cloutier et al., 2009; Cloutier, 2010), (2) juveniles are characterised by a partial body squamation (Cloutier, 2010), and (3) adults show complete body squamation (Cloutier, 2010). Histological data were gathered from transverse ground sections of complete specimens of two “early” juveniles, 10 “late” juveniles, and five adults (Chevrinais, Sire & Cloutier, 2017, see protocol). Elemental composition analyses were performed on two larval specimens, one juvenile ground section and one adult specimen (Chevrinais, Balan & Cloutier, 2015, see protocol).

### Spectrometry

Skeletal structures were considered as mineralised when calcium and phosphorus were recorded in proportion close to the hydroxyapatite composition (P-Ca%wt ratio around 1:2) (Dorozhkin & Epple, 2002). A small amount of calcium coupled with a high amount of carbon and no phosphorus was interpreted as calcified cartilage (Chevrinais, Balan & Cloutier, 2015). When a structure was mainly composed of carbon, it was interpreted as largely composed of collagen and identified as non-calcified cartilage.

### Developmental sequence and trajectory

Continuous (length of skeletal elements and distances among elements (Fig S1)) and discrete data (presence/absence of anatomical structures (Fig S2)) were collected on 178 specimens belonging to a growth series (29 larvae: 4.5–17.49 mm; 71 juveniles: 12.71–33.29 mm; 78 adults: 21.64–52.72 mm) (Chevrinais, Cloutier & Sire, 2015). The developmental (chondrification and ossification) sequences (i.e., the relative timing and order of skeletal events through ontogeny (Grünbaum, Cloutier & Vincent, 2012)) of *Triazeugacanthus* were reconstructed for 34 elements using 178 specimens. A developmental (ossified) trajectory (i.e., the cumulative addition of elements through ontogeny (Grünbaum, Cloutier & Vincent, 2012), also known as a maturity curve or bone maturity (Cloutier, 2010)) for *Triazeugacanthus* was reconstructed based on the ossification sequence.

Inter-individual variation in developmental sequence has been reported in developmental studies of living organisms (Colbert & Rowe, 2008; Maxwell, 2008; De Jong et al., 2009; Fischer-Rousseau, Cloutier & Zelditch, 2009). Here, we developed a reliability estimate (RE) (Text S1) calculated for each structure by dividing the actual number of specimens having an anatomical structure by the number of specimens expected to have this structure (i.e., number of specimens longer (in terms of TL) than the smallest specimen that displays the structure). The RE is calculated for each event because non-developmental sources of variation (e.g., taphonomic alteration, preservational position of the specimen) have the potential to alter differentially certain anatomical structures in fossilised ontogenies.

### Statistics

**Figure 2:**
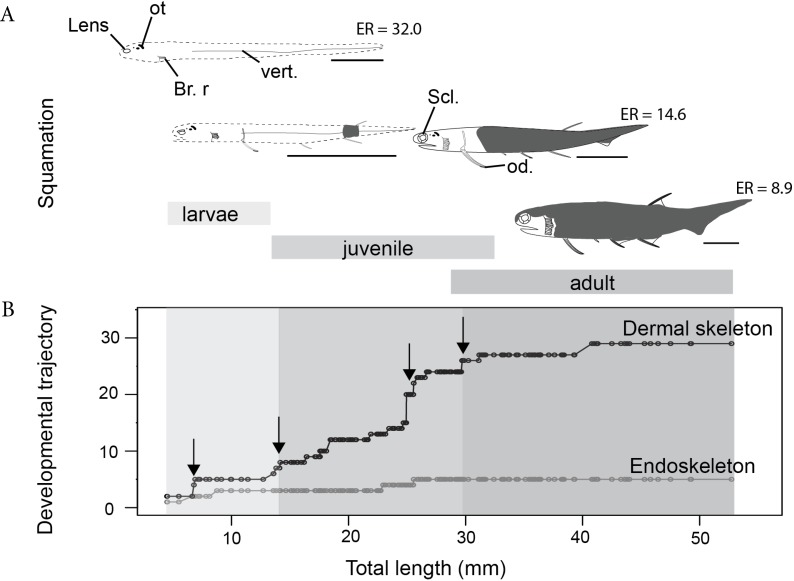
The development of the Late Devonian “acanthodian” *Triazeugacanthus affinis*. (A) reconstructions of ontogenetic stages focusing on squamation, and (B) developmental trajectory of endoskeleton and dermal skeleton. *N* = 178. From left to right, early larvae (scale bar = 1 mm), early juvenile, late juvenile, and adult reconstructions. Squamation development (dark grey) (Chevrinais, Sire & Cloutier, 2017). (Br. r), branchiostegal rays; (od.), paired fin odontode; (ot), otoliths; (Scl.), sclerotic bones; (vert.), vertebral elements. ER is for Elongation Ratio. Arrows indicate thresholds. Scale bars = 5 mm.

To characterize the growth of individual skeletal elements and shape changes during ontogeny, linear regressions between log_10_-transformed measurements and log_10_-transformed total length (log_10_TL) have been calculated for individual ontogenetic stages and for combined stages. Principal component analyses (PCA) on variance–covariance matrices of five log_10_-transformed measurements were performed for juveniles, adults and the combined dataset (Chevrinais, Cloutier & Sire, 2015). To measure continuous global body shape changes during growth, an elongation ratio was also calculated as a ratio of total length to body depth (Katz & Hale, 2016). Body depth was measured at the level of the dorsal fin spine, an anatomical element that could be identified in larval, juvenile and adult specimens (Fig. 2A). A high elongation ratio means that the body is very elongated. Comparison of elongation ratios among groups was performed using the non-parametric Kruskal–Wallis test and Tukey’s multiple comparisons test. Non-parametric tests were used because of the non-normality of the data. All statistical analyses were done with R 3.0.2.

**Figure 3:**
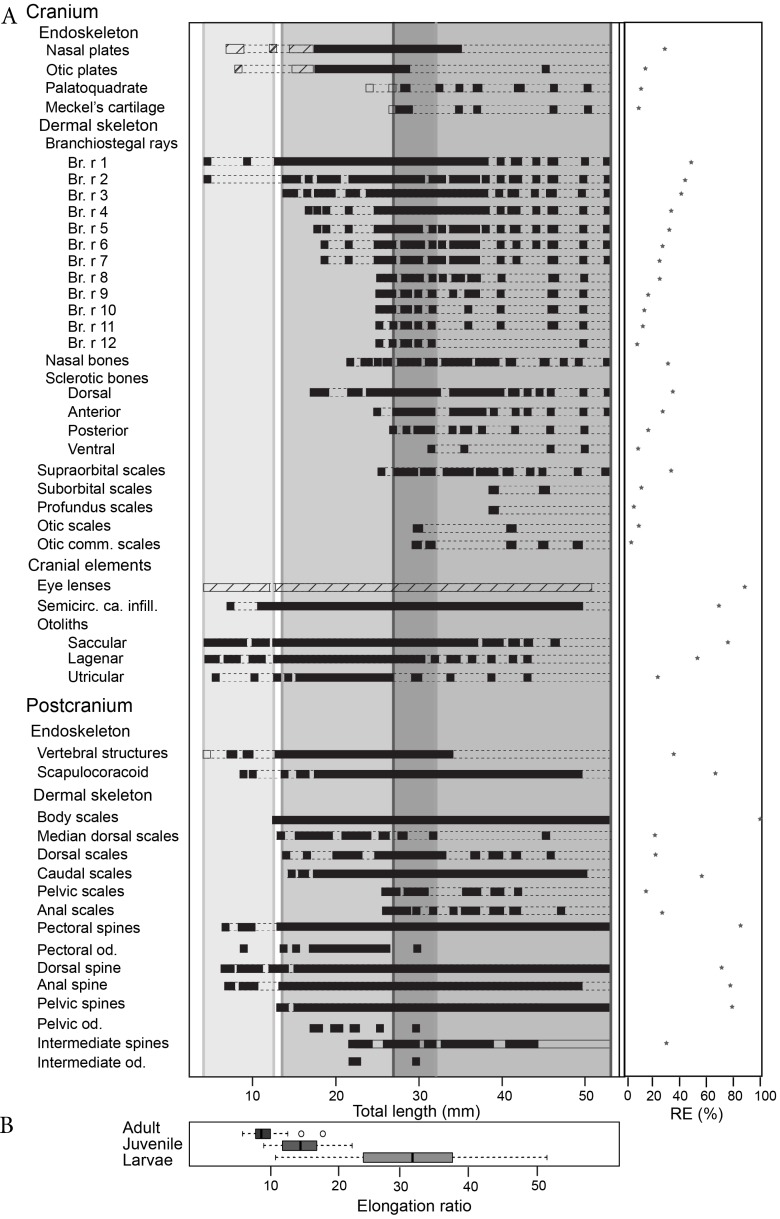
*Triazeugacanthus* developmental sequence (A). Light grey background, larvae; medium grey background, juveniles; dark grey background, adults; empty boxes, presence of a structure without information on the chemical composition; horizontal dashed lines, putative presence of a structure; full boxes, presence of a mineralised structure; stars, reliability index (*x* axis). (Br. r), branchiostegal rays; (od.), odontode; (Semicirc. ca. infill.), semicircular canal infilling. (B) Elongation ratios (RE) for larvae, juvenile and adult specimens.

## Results

The sequence of cumulative appearances of skeletal elements (based on 178 specimens of *Triazeugacanthus*) shows a developmental trajectory with periods of gradual or rapid change (i.e., thresholds) intercalated with periods of slow anatomical change (i.e., steps) (Fig. 2B). Endoskeletal elements are poorly represented in the sequence, especially because the scale coverage starts in early juveniles, hiding internal elements (Fig. 3A). Early in the larval period, a threshold occurs at 7 mm TL (i.e., development of neurocranial and vertebral elements, and pectoral, anal and dorsal fin spines). This threshold is followed by a step of slower development between 8 and 13 mm TL (Fig. 2B). The transition between the larval and juvenile periods is characterized by another threshold at 13 mm TL synchronously with the initiation of squamation. The juvenile period is characterised by extensive gradual addition of elements (more than 10 events developed over a period of 18 mm of growth) followed by a threshold at 29 mm TL. This threshold, coupled with the completion of the squamation, determines the transition between juveniles and adults. The adult stage shows a long step from 30 mm TL onward.

### Larvae (4.5–17.49 mm TL)

**Figure 4:**
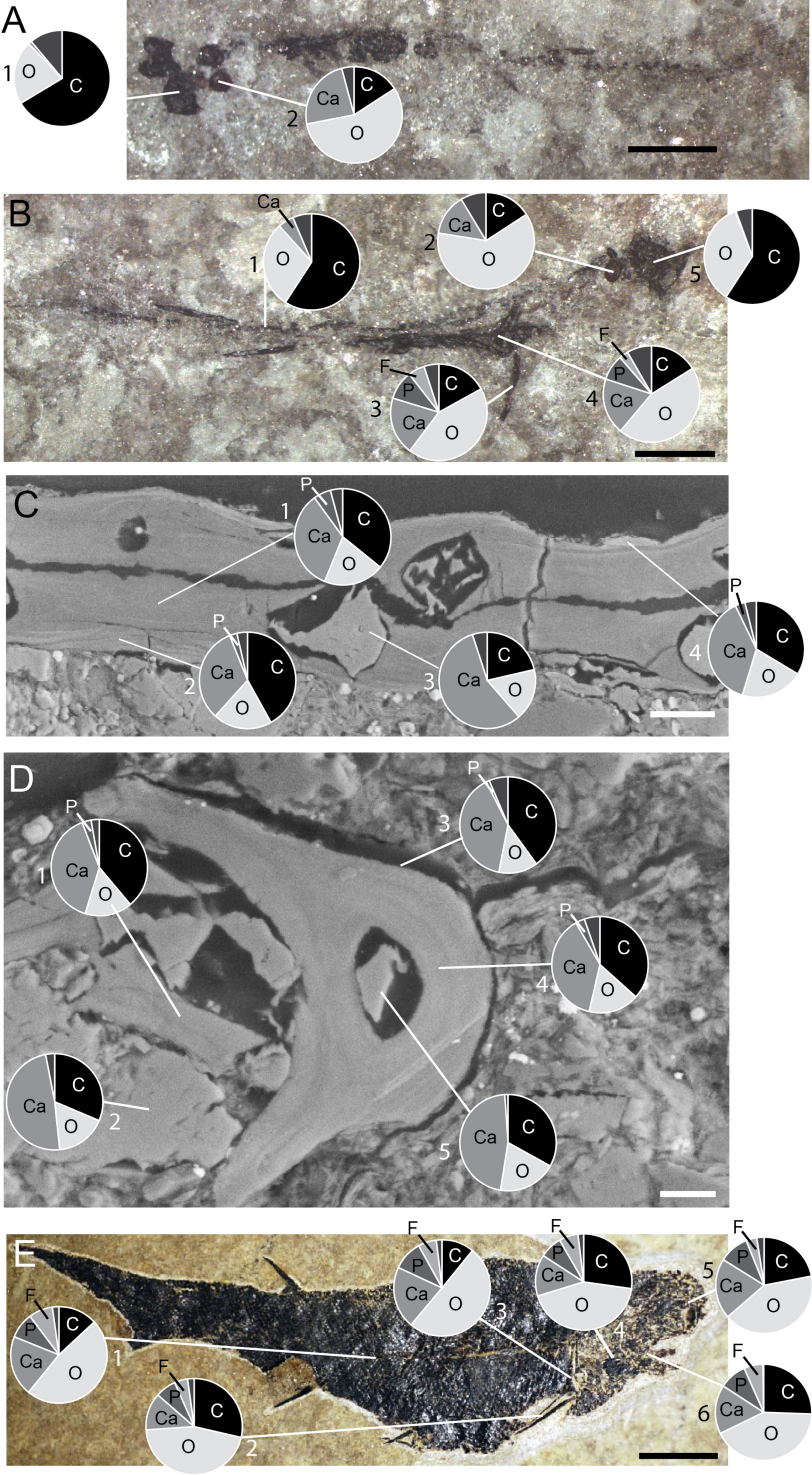
*Triazeugacanthus* EDS X-ray punctual microanalyses. Pie charts represent the relative percentage of main chemical elements. (A) MHNM 03-440. Eye lenses (1) and otoliths (2). (B) MHNM 03-440. Eye lenses (5), otoliths (2), scapula (4), pectoral spine (3) and vertebral structures (1). (C) MHNM 03-398. Juvenile endoskeleton (3) and scale inner (1) and outer layers (2, 4) from transverse sections. (D) MHNM 03-398. Juvenile endoskeleton (1, 2, 5) and anal spine inner (4) and outer layers (3). (E) MHNM 03-1497. Adult sclerotic bone (5), palatoquadrate (6), branchiostegal rays (4), scapula (3), pectoral spine (2) and scales (1). Scale bars = 1 mm in (A, B); 20 µm in (C), 10 µm in (D) and 5 mm in (E). Photo credit: Marion Chevrinais.

The first developmental stage available for *Triazeugacanthus* is the larval period; no embryonic specimens have been identified yet. The general body shape is relatively filiform; the average larval elongation ratio is 32 (Fig. 3B). Larval specimens are preserved dorso-ventrally. Paired eye lenses (*RE* = 88.2%), four otoliths (two hemispherical bean-shaped saccular otoliths and two ovoid intermediate lagenar otoliths (*RE* > 50%)), branchiostegal rays 1 and 2 and a series of vertebral elements (*RE* = 35%) are developed even in the smallest larvae (4.5 mm TL, MHNM 03-94). The successive crescent-shaped elements of carbonaceous composition (Fig. 4B1) represent most likely vertebral elements rather than the notochord. These vertebral elements are first recognized at the level of the dorsal and anal fin spines. Amorphous organic matter from the digestive tract obliterates their presence anterior to the dorsal fin. Cartilaginous neurocranial elements develop at 5.5 mm TL (nasal plates, *RE* = 28%) and at 6.5 mm TL (otic plates, *RE* = 11%). The two smallest spherical, utricular otoliths formed subsequently at 5.5 mm TL (*RE* = 23%). Associated with the presence of otoliths, granular semicircular canal infillings are recorded at 7 mm TL (*RE* = 69.2%) (Fig. 3A).

The appearance of fin spines is coupled with a lateral preservation of the specimens. Fin spines formed sequentially during the larval stage (Figs. 1 and 2): pectoral (*RE* = 85.1%) and dorsal (6.8 mm TL; *RE* = 70.1%), anal (6.9 mm TL; *RE* = 76.9%), and pelvic spines (14 mm TL; *RE* = 78.9%). The early development of the pectoral spines is accomplished by the addition of odontodes (as defined by Ørvig, 1977) at the distal extremity of the anterior ridge of the spines (9 mm TL) (Figs. 2,4A–4F). However, the precise number of odontodes is difficult to determine in the larval stage because of the fragile nature of these elements. The mineralised scapulocoracoid (8.8 mm TL; *RE* = 66.8%) (Figs. 4B4 and 4E3, Table S2) and fin spines display (Figs. 4B3, 4D4 and 4E2, Table S2) amounts of calcium and phosphorus superior to 19% wt and 8% wt, respectively. Still at 8.8 mm TL, vertebral structures show the presence of calcium (Fig. 4B1, Table S2). Despite the fact that axial and appendicular endoskeletal elements are already mineralised, neurocranial elements mineralised only at about 17 mm TL (Fig. 3A).

### Juveniles (12.71–33.29 mm TL)

The juvenile period is primarily characterized by the development of the dermal skeleton. Body shape is becoming slightly stockier; the average juvenile elongation ratio is 14.6 (Fig. 3B). Body squamation appears at 12.7 mm TL (*RE* = 100%), as a single small patch of primordium scales develop below the dorsal fin spine. Median dorsal scales develop anteriorly to the patch of body squamation at 13.8 mm TL (Fig S2). Their anterior position and their morphology suggest that median dorsal scales develop faster than body scales. The first scales associated with the fin webs are recorded almost simultaneously at the base of the dorsal fin (13.8 mm TL; *RE* = 21.4%) and at the base of the hypochordal lobe of the caudal fin (14.1 mm TL; *RE* = 55.7%). Body scales extend posteriorly to the caudal extremity and anteriorly reaching the region of the pectoral fins at 21 mm TL, which shows the transition between early and late juveniles. Subsequently, fin web scales develop proximo-distally in the pelvic (*RE* = 14.8%) and anal fins (25.6 mm TL; *RE* = 26.1%) (Fig. 3A, Fig S3). Within each web, scales are organized in adjacent rows in which the smallest scales are found distally. There is no indication of ceratotrichia in the fin webs. Cranial sensory line scales develop first at 25.9 mm TL with the supraorbital scales (*RE* = 33.3%), followed at 29.7 mm TL by the otic commissure and otic sensory line scales (*RE* = 3.6% and 9.1%, respectively).

Among the four sclerotic bones, the dorsal and anterior ones develop at 17.2 mm TL (*RE* = 34%) and 24.7 mm TL (*RE* = 27%), respectively. Branchiostegal rays 3 to 12 develop successively, from dorsal to ventral, through the juvenile period from 14 to 24.9 mm TL (Fig. 3A). Nasal bones form at 23.5 mm TL (*RE* = 33%). The last endocranial elements to develop are the mandibular elements, the palatoquadrate (23 mm TL; *RE* = 10.1%) forms slightly before the Meckel’s cartilage (25.6 mm TL; *RE* = 9.1%) (Fig. 3A).

**Figure 5:**
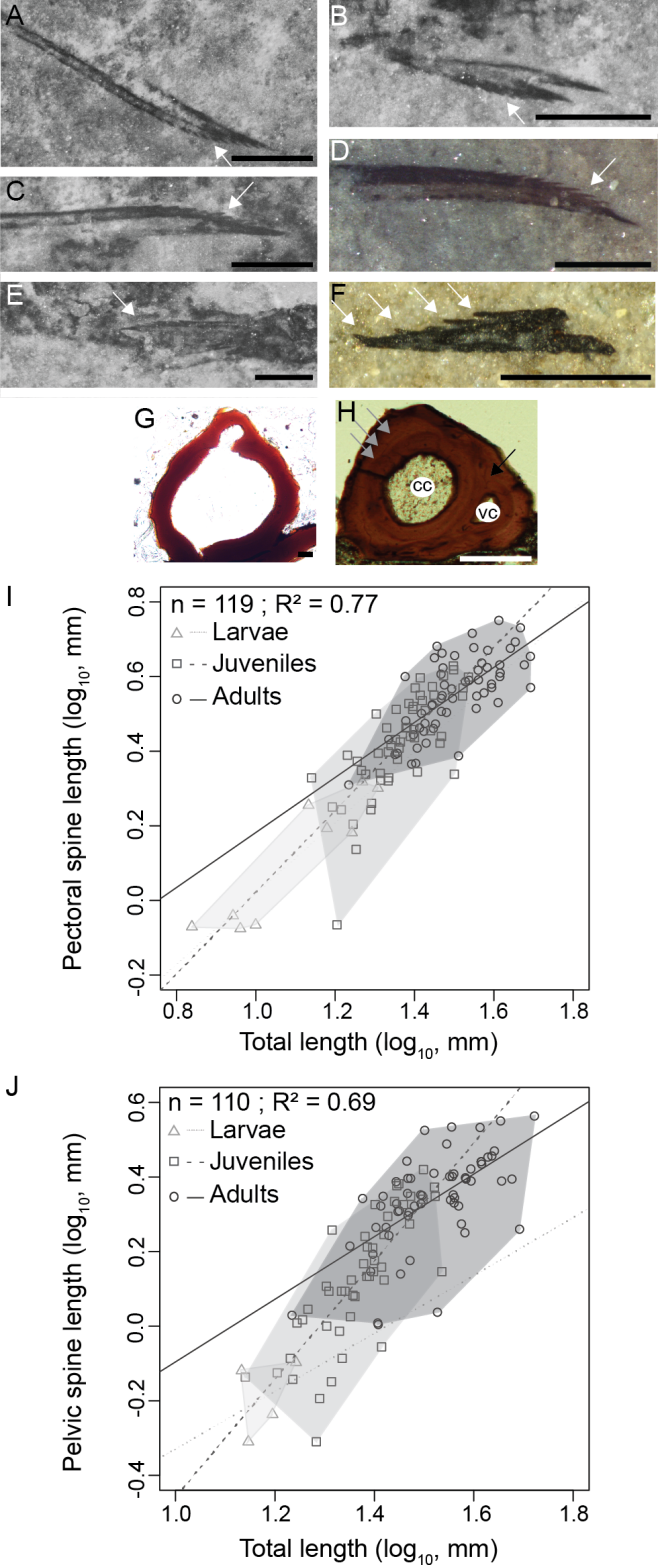
*Triazeugacanthus* paired fin spines. (A) MHNM 03-740. Left pectoral. (B) MHNM 03-740. Pelvics. (C) MHNM 03-740. Right pectoral. (D) MHNM 03-1985. Pectoral. (E) MHNM 03-210. Pelvics. (F) NMS 2002.59.15. Pectoral. (G) MHNM 03-701. Juvenile anal spine transverse section. (H) MHNM 03-2620. Adult anal spine transverse section. (I) Pectoral spine length and TL relationship. (J) Pelvic spine and TL relationship. (A–H) White arrows indicate odontodes, grey arrows growth lines and black arrow osteocyte cavities. Anatomical abbreviations: (cc), canal cavity; (vc), vascular canal cavity. Scale bars = 0.5 mm in (A–F), 20 µm in (G), 100 µm in (H). Photo credit: Marion Chevrinais.

The intermediate fin spines are the last spines to develop at 22 mm TL (*RE* = 30%). However, the presence of these spines is rare even in well-preserved adult specimens (Fig. 3A). Three odontodes are recorded on the intermediate fin spines at 22 mm TL. Denticles are still observed on the pectoral (six to ten odontodes from 18 to 25 mm TL specimens) and are recorded for the first time in pelvic (four to seven odontodes from 17 to 22 mm TL specimens) fin spines. These odontodes are also visible in ground sections of juvenile spines (Fig. 5G, top). Tissues composing the spines are difficult to observe in juveniles likely due to a poorly differentiated early stage of development (Fig. 5G); however, spine tissues are highly mineralised and contain a few cell cavities and tubules.

### Adults

The adult stage shows the completion of skeletogenesis (39 mm TL) and of the mineralisation (at least at 45 mm TL). The adult body shape is stocky; the average adult elongation ratio is 8.9 (Fig. 3B). The squamation is completed through the formation of the scales in the dorsal region of the head at around 26 mm TL. Cranial sensory line scales continue to develop with the profundus sensory line and the suborbital sensory line scales at 39.1 mm TL (*RE* = 5.6% and 11.1%, respectively). The development of the cranial sensory lines is similar to the main postero-anterior direction of the body squamation and also shows a dorso-ventral direction of progression. The lateral line canal is visible along the flank as a small space between two rows of scales, at mid-height, in the anterior part of the body (at least at 45 mm TL). In the posterior part of the body, the lateral-line canal is less visible than anteriorly and the scales seem to be closer to each other (Fig S2). The development of skeletal elements is completed by the formation of branchiostegal rays 3 to 12 (25 mm TL) and the ventral sclerotic bones (31 mm TL) (Fig. 3A, Table S1).

Scales continue to develop proximo-distally on the pelvic and anal fin webs. Consequently, fin spines are associated with scaled fin webs in the pelvic, anal and dorsal fins (Fig S3). Spine odontodes are recorded until the late juvenile/early adult stages; up to ten odontodes have been counted on a 0.84 mm long pectoral spine (Fig. 5C, MHNM 03-740). As the spine tissues get thicker, the individual odontodes become merged with the spine and therefore are not visible in adults. The histology of adult spines reveals mesodentine, with odontocyte cavities surrounding a large central vascular cavity that most likely housed the primary vascularization (Fig. 5H). A smaller vascular cavity, located dorsally to the central vascular cavity, is also present including a highly calcified tissue (Fig. 3D). The smallest vascular cavity seems to form later in spine ontogeny, after the formation of the central cavity (Fig. 5G). The tissue forming the boundary between the two cavities seems to develop secondarily (Figs. 5G and 5H). At least five growth zones are present in the ground section of the largest sectioned specimens.

### Ontogenetic trends

Shape variation is recorded through ontogenetic stages (Figs. 2A, 3B). Comparison of elongation ratios in larvae, juveniles and adults indicates a decrease in the elongation ratio from larvae to adults (Fig. 3B). The anterior part of the body is more elongated in early ontogeny (Fig. 2A, see reconstructions). Furthermore, the range of values and the standard deviation (sd) are higher for larvae (10.5 to 51; *sd* = 14.5) than for juveniles (8.7 to 22; *sd* = 3.7) and adults (5.5 to 17; *sd* = 2) (Fig. 3B). Body shape is thicker and less elongated than described previously (Gagnier, 1996).

The relationships between the length of skeletal elements and TL display different growth rates among ontogenetic stages (Figs. 5I and 5J). Linear regressions between lengths of fin spines and TL are significant (Figs. 5I and 5J); pectoral fin spines: *R*^2^ = 0.77, *p* < 2.2*e*^−16^; pelvic fin spines: *R*^2^ = 0.69, *p* = 2.2*e*^−16^). Slopes of the linear regressions between the growth of the pectoral spine and TL and the pelvic spine and TL increase from larvae (pectoral: slope = 0.92, *R*^2^ = 0.81, *p* = 2.1*e*^−10^) to juveniles (pectoral: slope = 1.04, *R*^2^ = 0.55, *p* = 1.6*e*^−10^; pelvic: slope = 1.64, *R*^2^ = 0.64, *p* = 2.4*e*^−11^) and decrease in adults (pectoral: slope = 0.76, *R*^2^ = 0.53, *p* = 3.2 = 3*e*^−4^; pelvic: slope = 0.72, *R*^2^ = 0.32, *p* = 9.3*e*^−8^) showing that most of the differential growth occurred during the juvenile period. Those results coupled with growth of individual skeletal elements (Figs. 5I and 5J) suggest that an allometric tendency is observed in juvenile pectoral and pelvic spines growth.

PCA loadings show that the principal source of body shape variation (65% of the variation) remains in pelvic to anal spines distance in juveniles (allometric coefficient = 4.5) and in anal to dorsal spines distance in adults (allometric coefficient = 2.2) arguing for positive allometry of the region between the pelvic fins and the dorsal fins. Those results, coupled with individual skeletal elements growth (Figs. 5I and 5J) suggest that an allometric tendency characterized the juvenile period.

## Discussion

Based on 178 specimens, morphological, histological, and chemical changes during the ontogeny of *Triazeugacanthus affinis* were extensively analysed for the first time in a Palaeozoic vertebrate species showing that: (1) the sequence of appearance of endoskeletal and exoskeletal elements follows a developmental trajectory with an alternation of thresholds and steps, (2) skeletal systems have specific directions of formation, (3) skeletal elements mineralize progressively during the larval and juvenile stages to be completed during the adult stage, (4) positive allometry is recorded during the juvenile stage, (5) the elongation ratio decreases during ontogeny, and (6) the variation in body shape decreases from larvae to adults during ontogeny. Besides body size, we used four ((1) the degree and timing of ossification, (2) the degree of squamation, (3) the allometric growth of individual skeletal elements, and (4) body proportions) out of the six criteria proposed by Cloutier (2010) to characterize immature specimens.

### Developmental trajectory

Developmental trajectory provides an overview of the critical periods during ontogeny. A succession of thresholds (periods with the appearance of numerous elements within a short period of time) and steps (periods of slower development) during skeletogenesis has already been documented during the ontogeny of extinct osteolepiforms (Cloutier, 2010) and living actinopterygians (Balon, 2002; Belanger, Balon & Rawlings, 2010). In living fishes, a saltatory pattern of development displays an alternation of thresholds and steps, where thresholds are associated with major physiological (e.g., endogenous to exogenous feeding), behavioural (e.g., passive to active movements), and ecological (e.g., passive to active predation, habitat changes) changes (Balon, 2001; Balon, 2002). We used the thresholds to delimit three periods in *Triazeugacanthus* ontogeny, larval, juvenile and adult. Our delimitation of the three periods slightly differs from our previous interpretation based mainly on the squamation extent (Chevrinais, Balan & Cloutier, 2015; Chevrinais, Cloutier & Sire, 2015). The main difference between these two hypotheses pertains to the overlap between stages. The gradual period in *Triazeugacanthus* seems to concur with the progression of squamation. The gradual change could either reflects (1) the “noise” of individual variation (as suggested also by the presence of the overlap between ontogenetic stages, Figs. 2A and 3A) (Balon, 2001), (2) an underestimation of the threshold due to the low number of skeletal elements (34 elements at maturity), or (3) a true ontogenetic compensation where the energy is focused on global exoskeletal development rather than the formation of specific structures. Since saltatory ontogeny has been recognized in living actinopterygians, extinct sarcopterygians and “acanthodians”, it is suggested that it might represent a generalised gnathostome pattern inherited at least from Ordovician time.

During the ontogeny, some internal structures were hidden by the development of external structures (e.g., scales) which potentially could bias the developmental trajectory. Structures showing a good reliability (*RE* > 50%) reflect adequately the growth in *Triazeugacanthus*, whereas endoskeletal elements (e.g., neurocranium, vertebral structures), elements covered by scales (e.g., otoliths), or kinetic structures (e.g., branchiostegal rays, lower jaw) show a relatively low reliability in both *Triazeugacanthus* and *Triazeugacanthus* sister species, *Lodeacanthus* (Upeniece, 1996; Hanke & Davis, 2012; Chevrinais, Sire & Cloutier, 2017) (Tables S1 and S4). These elements were subject to loss more easily than structures ankylosed or having complex sutures.

*Triazeugacanthus* shows no metamorphosis as suggested by the continuity in the growth of individual elements as well as by the progressive shape variation among ontogenetic stages; this “acanthodian” has a direct development (Fig. 2). Body proportions changed during ontogeny mainly during the juvenile period (Cloutier, 2010). *Triazeugacanthus* is more elongate at the larval stage than at the adult stage as reflected by the elongation ratio. Such a shape variation is also present in the majority of actinopterygians (Katz & Hale, 2016) and chondrichthyans (Table S3). Change in body proportions through ontogeny is one of the criteria for recognition of fossil ontogenies (Cloutier, 2010), such as in the early actinopterygian ‘*Elonichthys*’ *peltigerus* (Schultze & Bardack, 1987).

### Cranium

Among the anatomical features showing early differentiation during the ontogeny of living vertebrates, cranial systems (i.e., vision, breathing, feeding, and equilibrium or balance) develop first, allowing the fish larvae to perceive and interact with the environment (Osse et al., 1997; Wyffels, 2009); evidently postcranial support is also necessary to react to these initial stimuli. The smallest larval specimen of *Triazeugacanthus* (*TL* = 4.5 mm) already displays eye lenses, otoliths (saccular and lagenar), two branchiostegal rays and vertebral structures. The early appearance of the eye lenses (Fig. 3A) is congruent with that observed in “acanthodians” (Heidtke, 1990; Upeniece, 2011), extinct and living chondrichthyans (Wyffels, 2009; Sallan & Coates, 2014), and osteichthyans including tetrapods (Schoch, 2006; Hall, 2008; Cloutier et al., 2011). In terms of body proportions, the eyes being proportionally larger in immature than adult specimens is considered as a recurrent growth pattern in osteichthyans (Schultze, 1984; Schultze & Bardack, 1987; Cloutier, 2010). Nevertheless, this pattern is also present in other gnathostomes such as “placoderms” (Werdelin & Long, 1986; Trinajstic & Hazelton, 2007; Cloutier, 2010) and living chondrichthyans (Wyffels, 2009), but also in agnathans such as Petromyzontiformes (Vladykov & Kott, 1980; Chang et al., 2014). However, in the living lamprey, the diameter of the eye could also increase after metamorphosis and during the adult stage (Youson, 1980), because at metamorphosis the eyes come closer to the epidermal surface and consequently appear larger (Youson, 1980). *Triazeugacanthus* eye lenses show significant differences in growth during ontogeny (Chevrinais, Cloutier & Sire, 2015, Fig. 2A, the slopes of linear regressions are different between larvae, juveniles and adults), thus showing a typical gnathostome pattern or potentially an early vertebrate one.

Associated with the eyes of teleosts, the anterior and posterior ossicles ossified from a cartilage ring surrounding the ocular globe (Franz-Odendaal & Vickaryous, 2006). In amniotes, sclera ossicles (homologous or not to those of fish) form either in a clockwise manner starting ventrally or in an alternate manner (posterior, anterior, dorsal and ventral) (Zhang et al., 2012). The four sclerotic bones (sclera ossicles) of *Triazeugacanthus* develop sequentially (dorsal, posterior, anterior and ventral) in juveniles and early adults. Sequential development of sclerotic bones is known in other “acanthodians” (Heidtke, 1990; Upeniece, 2011). The variable number of sclerotic bones (even in closely related species of “acanthodians”) and the variation in their developmental pattern indicate a high disparity among gnathostomes, necessitating further comparative studies.

Three pairs of otoliths are known in acanthodiform “acanthodians” (Schultze, 1990). Saccular and lagenar otoliths develop during the embryonic stage in *Danio rerio* (Riley & Moorman, 2000) and *Polypterus senegalus* (Bartsch, Gemballa & Piotrowski, 1997). As in *D. rerio* (Haddon & Lewis, 1996; Riley & Moorman, 2000), the saccular and lagenar otoliths of *Triazeugacanthus* develop first, followed by the utricular. In *D. rerio*, and most likely in *Triazeugacanthus*, the saccular and lagenar otoliths develop early during the embryonic stage. The record of growth lines in the otoliths in *Triazeugacanthus* (Chevrinais, Cloutier & Sire, 2015, Annexe II) is congruent with observations already made by Gagnier (1996), who reported the presence of concentric growth zones enclosing minor secondary order zones. Three pairs of otoliths are recorded early in the ontogeny of *Acanthodes lopatini* (Beznosov, 2009) and *A. bronni* (Heidtke, 1990) (in which statoconia are followed by three otoliths in ontogeny) and osteichthyans, whereas they are absent in chondrichthyans (Schultze, 1990). Schultze (1990) considered the presence of three pairs of otoliths as a synapomorphy shared by “acanthodians” and osteichthyans. Recent phylogenetic analyses of gnathostomes did not use otolith characters (Brazeau, 2009; Davis, Finarelli & Coates, 2012; Burrow et al., 2016; Chevrinais, Sire & Cloutier, 2017) and show “acanthodians” as stem chondrichthyans (Chevrinais, Sire & Cloutier, 2017); thus otoliths could represent a condition shared by some “acanthodians”, some chondrichthyans and some osteichthyans, or some “acanthodians” could be related to osteichthyans and others to chondrichthyans, with statoconia considered plesiomorphic for gnathostomes (Schultze, 1990).

Branchiostegal rays develop relatively early in *Triazeugacanthus*, considering they belong to the exoskeleton which usually develops later; early development of branchiostegal rays has been also documented in *Lodeacanthus* (Table S4) (Upeniece, 2011) and *Acanthodes* (Zajic, 2005). The presence of elements covering externally the hyoid and branchial apparatus suggests that branchial respiration (versus skin respiration) is already acquired and efficient early in ontogeny. Bony branchiostegal rays are also present in most basal actinopterygians (Arratia & Schultze, 1990; Cloutier & Arratia, 2004) whereas they are missing in chondrichthyans. Arratia & Schultze (1990) reported that actinopterygian branchiostegal rays are homologous to that of “acanthodians”. Based on a topographic criterion the condition in both taxa is similar. Furthermore, the directionality of formation for these rays is similar in both taxa; posterodorsal to anteroventral for actinopterygians (Arratia & Schultze, 1990) and dorsal to ventral in *Triazeugacanthus*. External gills have not been reported in “acanthodians” either because these structures (1) have a weak potential for fossilisation, (2) are absent or (3) are only present in very early larval stages. Wyffels (2009) considered the presence of external gill filaments as plesiomorphic for chondrichthyans, while these structures are also present in basal actinopterygians (Bartsch, Gemballa & Piotrowski, 1997), lungfishes and amphibians (Fritzsch, 1990).

Jaw development occurs early in ontogeny of chondrichthyans and osteichthyans (Bemis & Grande, 1992; Grande & Bemis, 1998; Summers, Ketcham & Rowe, 2004; Wyffels, 2009; Cloutier et al., 2011). Jaws are attached to the neurocranium early in ontogeny (Wagemans, Focant & Vandewalle, 1998) and show a mineralisation allowing a good preservation (Grande & Bemis, 1998). In both *Triazeugacanthus* and *Lodeacanthus*, the presence of jaws is recorded relatively late and is poorly reliable (Table S4) (Upeniece, 2011). In contrast, the mineralisation of the jaws in *Acanthodes* is completed early in ontogeny (Zidek, 1985). The poor preservation of jaws in *Triazeugacanthus* and *Lodeacanthus* and the relatively large time range of formation and mineralization observed in other acanthodiforms suggest that jaw bones may be weakly attached to the neurocranium or weakly preserved (due to the prismatic mineralization of these two pairs of elements) in acanthodiforms in comparison to the condition observed in chondrichthyans and osteichthyans.

In addition to the eye lenses, the larval neurocranium included the otic and nasal plates; chemical analyses revealed that they first chondrify before their mineralization. A general postero-anterior direction of formation has been suggested in the neurocranium of chondrichthyans (Summers, Ketcham & Rowe, 2004; Johanson et al., 2013) and in extinct and living actinopterygians (Schultze & Bardack, 1987; Grande & Bemis, 1998). This general postero-anterior direction of ossification was also found for the ossification of dermal cranial structures (e.g., scales, branchiostegal rays) in *Triazeugacanthus* and *Lodeacanthus*. However, an antero-posterior ossification of dermal bones is observed in basal actinopterygians (Schoch, 2006).

### Postcranium

#### Axial skeleton

The notochordal and vertebral elements develop early in fishes (Hall, 2008). Axial skeletal elements are rarely preserved in “acanthodians” and their developmental pattern is mostly unknown. Only one specimen of *Acanthodes sulcatus* (Lower Carboniferous) shows vertebral elements (e.g., neural and haemal arches) along the body (Miles, 1970). Although there is no indication of well-developed vertebral elements in adult *Triazeugacanthus*, cartilaginous precursors were documented in early larvae before their mineralization in later stages. The poor record of the “acanthodian” axial skeletons, even if arches are perichondrally ossified, is most likely due to the weak mineralization of the vertebral elements.

#### Paired fins

Gnathostomes are characterised by the presence of endoskeletal and/or dermal girdles supporting paired fins (Coates, 2003). “Acanthodians” show both endoskeletal and dermal pectoral girdles; however, acanthodiforms show only an endoskeletal pectoral girdle. In all acanthodiform ontogenies, a mineralised scapulocoracoid is the first element of the pectoral girdle to develop (i.e., *Triazeugacanthus* (this study), *Lodeacanthus* (Table S4) (Upeniece, 2011), *A. bronni* (Heidtke, 1990), *A. bridgei* (Zidek, 1985)). However, the ontogenetic occurrence of pectoral spines before the scapulocoracoids, might be explained by the presence of an undocumented cartilaginous precursor of the scapulocoracoids.

One of the main “acanthodian” characteristics is the presence of fin spines in front of each fin except the caudal fin (Denison, 1979; Miller, Cloutier & Turner, 2003; Brazeau & Friedman, 2014). *Triazeugacanthus* paired fin spines (pectoral, pelvic, and intermediate) are characterised by high RE with the exception of the intermediate spines. The late development of these intermediate spines as well as their low frequency of occurrence (32.4% of the specimens) could be interpreted as the presence of a sexual dimorphism. In gnathostomes, pectoral fin spines are only known in “acanthodians”, the “placoderm” *Macropetalichthys* (Denison, 1978), four osteichthyans (*Achoania*, *Guiyu*, *Psarolepis* and *Sparalepis* (Zhu, Yu & Janvier, 1999; Zhu & Yu, 2009; Choo et al., 2017)), basal chondrichthyans (*Doliodus problematicus* (Miller, Cloutier & Turner, 2003), *Wellerodus priscus* (Potvin-Leduc et al., 2011) and also suggested in *Antarctilamna prisca* (Miller, Cloutier & Turner, 2003)); however, none of them shows a developmental sequence as in *Triazeugacanthus*. The paired fin spines of *Triazeugacanthus* grow by distal accretion of odontodes. Small odontodes have also been observed in other acanthodiforms: pectoral, intermediate and pelvic fin spines in juvenile *Lodeacanthus* (Upeniece, 1996; Upeniece, 2011), and paired fin spines of the juvenile *A. lopatini* (Beznosov, 2009). In the so-called ‘juveniles’ of *Tetanopsyrus breviacanthias* (Lower Devonian), paired fin spines are completely formed by small odontodes (Hanke, Davis & Wilson, 2001). Histological composition of fin spines differs among “acanthodians” (Denison, 1979; Burrow et al., 2016). *Triazeugacanthus* shows the presence of mesodentine with two canals (likely vascular). The small vascular cavity develops after the large central cavity. Larger “acanthodians”, such as *Rhadinacanthus* and *Diplacanthus*, have the presence of trabecular mesodentine and high vascularisation (accessory pulp canals) in paired fin spines (Burrow et al., 2016). In these diplacanthiforms, growth zones have only been observed in the central part of the spines, close to pulp cavities (Burrow et al., 2016), thus differing from *Triazeugacanthus* where growth zones are present even in the periphery, close to the distal margin. In term of histology, *Triazeugacanthus* spine growth is similar to that of chondrichthyans (Maisey, 1979).

During ontogeny, the pectoral spines of *Triazeugacanthus* develop before the pelvic spines. Given that fin webs are absent, the spines are the only components of pectoral fins in *Triazeugacanthus*. The development of pectoral fins before pelvic fins is a pattern that has been repeatedly documented in chondrichthyans and osteichthyans (Ballard, Mellinger & Lechenault, 1993; Bartsch, Gemballa & Piotrowski, 1997; Didier, LeClair & Vanbuskirk, 1998; Joss & Longhurst, 2001; Mabee et al., 2002; Maxwell, Fröbisch & Heppleston, 2008; Riley, Cloutier & Grogan, 2017). This result supports the hypotheses that (1) pectoral fins appeared before pelvic fins during evolution (Coates, 1993; Coates, 1994; Larouche, Zelditch & Cloutier, 2017), and (2) pelvic fins are developmental duplicates from pectoral fins (Freitas, Zhang & Cohn, 2007).

### Scales

The completion of the squamation is one of the criteria defining the passage from the juvenile to the adult stage in gnathostomes (Cloutier, 2010). *Triazeugacanthus* squamation initiates in the region below the dorsal fin spine, and extends bidirectionally to completion in adults. Exhaustive comparison of this pattern has shown that the bidirectional pattern is common in “acanthodians” (Chevrinais, Sire & Cloutier, 2017) and teleosts (Sire & Arnulf, 1990). This squamation pattern might well represent a precursor condition to the unidirectional development of initial scales in chondrichthyans (Johanson, Smith & Joss, 2007). We agree with Johanson, Smith & Joss (2007) who considered that the presence of scale patterning maintained through ontogeny might be a synapomorphy of crown group gnathostomes.

**Table 1:** Similarities and differences of anatomical and developmental traits between *Triazeugacanthus*, osteichthyans and chondrichthyans. Detailed comparisons are given in the main text.

Anatomy	Chondrichthyans	*Triazeugacanthus*	Osteichthyans
Otoliths	Statoconia or one/two pairs	Three pairs	Three pairs
Branchiostegal rays	Absent	Present	Present
External gills	Present	Absent (or unknown)	Present/Absent
Vascular canal in spine	Present	Present	Present
**Development**			
Ontogeny	Not available	Saltatory	Saltatory
Eye lenses	Early ontogeny	Early ontogeny	Early ontogeny
Eye negative allometry	Yes	Yes	Yes
Sclerotic elements	Cartilage mineralization	Bone	Cartilaginous precursor
Order of sclerotic bones formation	Not available	Sequential	Sequential
Saccular and lagenar otoliths develop before utricular	Not applicable	Yes	Yes
Jaw development	Early embryonic development	Not available	Early embryonic development
Jaw mineralization	Early larval development	Late larval development	Early larval development
Prismatic mineralization	Present	Not available^a^	Absent
Cranial direction of mineralization	Postero-anterior	Postero-anterior	Postero-anterior
Branchiostegal direction of formation	Not applicable	Dorsal to ventral	Posterodorsal to anteroventral
Axial skeleton composition	Mineralized cartilage	Cartilaginous	Ossified
Origin of pectoral girdle	Endochondral	Endochondral	Endochondral and dermal
Pectoral fins develop before pelvic fins	Yes	Yes	Yes
Fin order	Pectoral - dorsal/anal - pelvic	Pectoral - dorsal/anal - pelvic	Pectoral - dorsal/anal - pelvic
Paired fin spine growth	Not available	Distal accretion of odontodes	Not available
Squamation direction	Postero-anterior	Bidirectional	Bidirectional
Lateral line canal	Not available	Antero-posterior	Antero-posterior
Elongation ratio	Highest in larvae	Highest in larvae	Highest in larvae

**Notes.**

a Irregularly patterned calcified cartilage (Burrow et al., 2016) and granular mineralization of the cartilage (Newman et al., 2014) have been recorded in “acanthodians”.

Scale cover and more specifically squamation development in *Triazeugacanthus* allows us to infer lateral line canal development (Fig S2). The presence of a gap between scales in the anterior part of the body and its absence in the posterior part suggest that the development of the lateral line canal constrains the squamation development and thus, occurs before the squamation is completed (Fig S2). However, in *Acanthodes*, scales develop in front of the anal spine, following the lateral line in the direction of the dorsal side of the head (Zidek, 1976; Forey & Young, 1985; Heidtke, 1990; Zajic, 2005; Upeniece, 2011). This suggests a close relationship between lateral line and squamation development. In *Triazeugacanthus*, the lateral line canal seems to develop antero-posteriorly and the squamation postero-anteriorly (Chevrinais, Sire & Cloutier, 2017) (such as in the actinopterygian *Danio rerio* (Ghysen & Dambly-Chaudière, 2004; Sire & Akimenko, 2004)). This similarity between *Triazeugacanthus* and *D. rerio* could corroborate the hypothesis that lateral line canal and squamation develop independently in gnathostomes.

## Conclusion

The exhaustive description of the ontogeny of *Triazeugacanthus affinis* from 178 specimens ranging from 4.5 to 52 mm TL gives the opportunity to describe development of individual skeletal structures (mainly dermal ones), of fin spines and squamation. Developmental trajectory shows the alternation of steps and thresholds comparable with other gnathostomes and showing that *Triazeugacanthus* ontogeny represents the oldest model for the study of development in gnathostomes, thus potentially representing plesiomorphic characters for gnathostomes (Table 1, see common characters of the three groups). Despite a hypothesized phylogenetic position of “acanthodians”, placing them as stem chondrichthyans in phylogenetic analyses that do not include developmental characters (Burrow et al., 2016; Chevrinais, Sire & Cloutier, 2017), some developmental characteristics of *Triazeugacanthus* are shared with osteichthyans rather than chondrichthyans (e.g., development of cranial elements such as otoliths) (Table 1). Thus, developmental data (e.g., appearance and development of skeletal elements through growth) represent understudied source of data, and have potential to be included in further phylogenetic analyses, notably for a better resolution of early vertebrate relationships.

##  Supplemental Information

10.7717/peerj.3969/supp-1 Supplemental Information 1 Supplementary figures, tables, and materials

## Institutional abbreviations

LDM: Latvijas Dabas Muzejs (Latvia)
MHNM: Musée d’Histoire Naturelle de Miguasha (Canada)
NHM: The Natural History Museum (United Kingdom)
NMS: National Museums of Scotland (Scotland)
UPMC: Université Pierre et Marie Curie (France)
UQAR: Université du Québec à Rimouski (Canada)
