# Supplementary material for: Unravelling the ontogeny of a Devonian early gnathostome, the “acanthodian” Triazeugacanthus affinis (eastern Canada)
Source: PeerJ. 2017 Oct 27;5:e3969. doi: 10.7717/peerj.3969 (PMC5661438; doi:10.7717/peerj.3969)
Supplement: Supplemental Information 1 [file peerj-05-3969-s001.pdf]

Unravelling the ontogeny of a Devonian early gnathostome, the "acanthodian" *Triazeugacanthus affinis* (eastern Canada)

Marion Chevrinais[1], Jean-Yves Sire[2] and Richard Cloutier[1]

[1]Laboratoire de Paléontologie et biologie évolutive, Université du Québec à Rimouski, Rimouski, Canada.

[2]CNRS-UMR 7138-Evolution Paris-Seine IBPS, Université Pierre et Marie Curie, Paris, France

*corresponding author: marion.chevrinais@uqar.ca

3 figures
4 tables
1 text

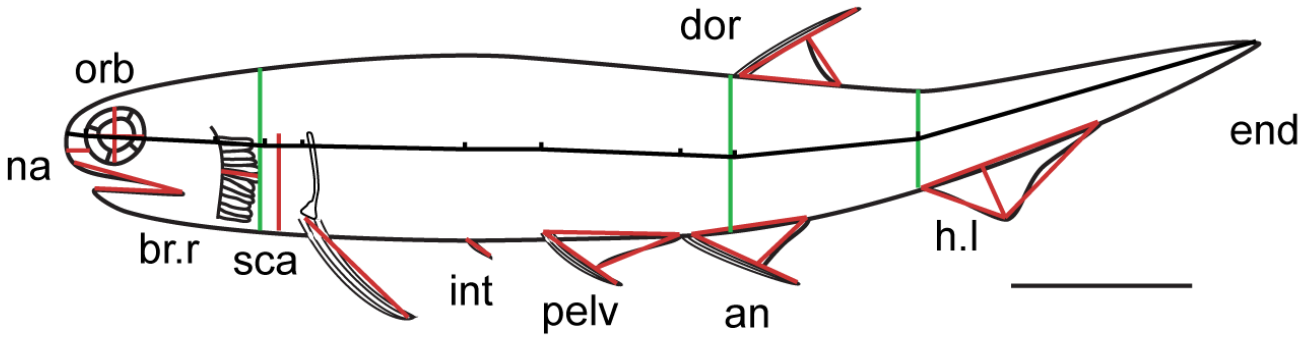

Figure S1. *Triazeugacanthus* skeletal structure measurements and distances. An: anal fin; br.r: branchiostegal rays; dor: dorsal fin; h.l: hypochordal lobe; na: nasal bones; orb: orbit; sca: scapulocoracoid; int: intermediate fins; pelv: pelvic fins; end: posterior extremity of the body. Scale bar = 5 mm.

Table S1. Raw data of presence/absence of skeletal elements in *Triazeugacanthus affinis* used for Reliability index calculation. White is for absence. Grey is for presence.

Specimen MHNM	Estimated TL (mm)	Eye lenses	Semicircular canals	Otoliths			Nasal plate	Palatoquadrate	Meckel's cartilage
				Saccular	Lagenar	Utricular			
03-94	4.5								
03-403	4.51								
01-260	5.5								
01-250	6.64								
03-843	6.79								
03-440	6.9								
03-1292	7.17								
03-440	7.29								
01-271	7.82								
03-403	8.12								
03-440	8.76								
01-275	9.14								
03-2729	9.94								
01-256	9.99								
03-1292	10.84								
01-253	11.4								
03-2684	12.71								
03-316	13.58								
01-230	13.6								
03-259	13.82								
03-622	14.1								
03-418	14.17								
03-1378	14.65								
01-230	15.1								

Continued on next page

Continued from previous page

Specimen MHNM	Estimated TL (mm)	Eye lenses	Semicircular canals	Otoliths			Nasal plate	Palatoquadrate	Meckel's cartilage
				Saccular	Lagenar	Utricular			
03-417	15.11								
03-1778	15.32								
03-2015	15.59								
03-890	15.67								
03-1515	15.98								
03-372	16.2								
03-1438	16.42								
03-2684	16.43								
03-1480	17.15								
03-398	17.2								
03-1204	17.22								
03-2756	17.47								
03-210	17.55								
03-1976	17.55								
03-319	17.58								
03-1897	17.7								
03-1408	17.9								
03-1252	18.1								
03-435	18.46								
03-654	18.55								
03-2033	18.86								
03-2001	18.91								
03-2015	19.2								
03-503	19.34								

Continued on next page

Continued from previous page

Specimen MHNM	Estimated TL (mm)	Eye lenses	Semicircular canals	Otoliths			Nasal plate	Palatoquadrate	Meckel's cartilage
				Saccular	Lagenar	Utricular			
03-1427	19.5	■	■	■	■	■	■		
03-1916	19.58	■	■	■	■				
03-2599	19.93	■	■	■	■	■	■		
03-1516	20.12	■	■	■	■		■		
03-401	20.14	■	■	■	■	■	■		
03-354	20.3	■	■	■	■				
03-1916	20.35	■	■	■	■	■	■		
03-1881	20.59	■	■	■	■	■	■		
03-1378	20.65	■					■		
03-1250	21.37	■	■	■	■	■	■		
03-433	21.55	■	■	■			■		
03-2684	21.57	■	■	■	■	■	■		
03-549	21.64	■	■	■	■				
03-978	21.64	■	■	■	■	■	■		
03-740	21.92	■	■	■	■	■			
03-531	22.43	■		■					
03-1961	22.48	■	■	■	■		■		
03-561	22.59	■	■	■	■	■	■		
03-1963	22.73		■						
03-1192	22.75	■	■	■	■	■			
03-2570	22.96	■	■	■	■		■		■
03-1515	23.15	■	■	■	■		■		
03-1205	23.47	■	■	■	■				
03-1833	23.75	■					■		
03-1962	23.79	■	■	■	■	■			
03-363	23.88	■							

Continued on next page

Continued from previous page

Specimen MHNM	Estimated TL (mm)	Eye lenses	Semicircular canals	Otoliths			Nasal plate	Palatoquadrate	Meckel's cartilage
				Saccular	Lagenar	Utricular			
03-1819	23.9								
03-813	23.9								
03-2480	24.05								
03-1236	24.25								
03-432	24.31								
03-2477	24.55								
03-916	24.7								
03-2483	24.86								
03-1985	24.93								
03-1509	24.95								
03-2440	25.12								
03-450	25.21								
03-558	25.3								
03-1879	25.3								
03-2629	25.51								
03-1847	25.53								
03-25	25.57								
03-1100	25.88								
03-1311	25.97								
03-2660	25.97								
03-220	26.16								
03-2025	26.27								
03-1538	26.28								
03-78	26.52								
03-235	26.69								
03-2654	26.73								

Continued on next page

Continued from previous page

Specimen MHNM	Estimated TL (mm)	Eye lenses	Semicircular canals	Otoliths			Nasal plate	Palatoquadrate	Meckel's cartilage
				Saccular	Lagenar	Utricular			
03-695	26.87								
03-108	27.58								
03-1406	27.79								
03-2030	27.84								
03-2487	27.88								
03-369	27.96								
03-773	28.13								
03-1295	28.2								
03-1496	28.2								
03-2589	28.36								
03-2613	28.62								
03-1969	28.73								
03-1306	28.79								
03-123	29.13								
03-176	29.2								
03-2630	29.29								
03-867	29.34								
03-699	29.36								
03-1994	29.37								
03-164	29.47								
03-1051	29.49								
03-2025	29.57								
03-2612	29.63								
03-551	29.71								
03-1909	29.72								
03-336	29.87								

Continued on next page

Continued from previous page

Specimen MHNM	Estimated TL (mm)	Eye lenses	Semicircular canals	Otoliths			Nasal plate	Palatoquadrate	Meckel's cartilage
				Saccular	Lagenar	Utricular			
03-1687	30.24	■		■					
03-217	31.12	■	■	■					
03-868	31.17	■							■
03-716	31.34	■		■					
03-2631	31.47	■	■	■			■		
03-1008	31.53	■	■	■	■		■		
03-1314	31.64	■	■	■					
03-55	31.71	■		■					
03-2589	32.42		■	■					
03-1550	33.1	■	■	■	■				
03-701	33.18	■	■	■	■	■	■		
03-1533	33.29	■	■	■	■				
03-38	33.64	■	■	■				■	■
03-530	33.7								
03-2614	33.74	■	■						
03-1798	34.34	■	■	■	■				
03-723	34.48	■							
03-88	35.12	■	■	■					
03-2620	35.44			■					
03-597	35.6	■							■
03-570	35.75								
03-1083	35.92								
03-98	35.93	■	■	■	■			■	■
03-452	36.19	■							
03-1233	36.29								
03-1215	36.35		■	■					

Continued on next page

Continued from previous page

Specimen MHNM	Estimated TL (mm)	Eye lenses	Semicircular canals	Otoliths			Nasal plate	Palatoquadrate	Meckel's cartilage
				Saccular	Lagenar	Utricular			
03-1031	36.4								
03-933	36.41								
03-1731	37.11								
03-388	37.6								
03-1545	38.21								
03-525	38.3								
03-2719	38.41								
03-971	39.27								
03-584	39.31								
03-2621	40.8								
03-534	40.98								
03-347	41.2								
03-2637A	41.2								
03-2761	41.29								
03-198	42.5								
03-529	43.28								
03-528	43.6								
03-139	43.8								
03-58	44								
03-1497	45.29								
03-715	45.8								
03-961	46.45								
03-1817	47.51								
03-1971	49.21								
03-2679	49.27								
03-1107	52.72								

Continued on next page

Continued from previous page

Specimen MHNM	Estimated TL (mm)	Otic plates	Vertebral structures	Scapulo-coracoid	Body scales	Nasal bones	Sclerotic bones				Branchiostegal rays	
							Dorsal	Anterior	Posterior	Ventral	1	2
03-94	4.5											
03-403	4.51											
01-260	5.5											
01-250	6.64											
03-843	6.79											
03-440	6.9											
03-1292	7.17											
03-440	7.29											
01-271	7.82											
03-403	8.12											
03-440	8.76											
01-275	9.14											
03-2729	9.94											
01-256	9.99											
03-1292	10.84											
01-253	11.4											
03-2684	12.71											
03-316	13.58											
01-230	13.6											
03-259	13.82											
03-622	14.1											
03-418	14.17											
03-1378	14.65											
01-230	15.1											

Continued on next page

Continued from previous page

Specimen MHNM	Estimated TL (mm)	Otic plates	Vertebral structures	Scapulo-coracoid	Body scales	Nasal bones	Sclerotic bones				Branchiostegal rays	
							Dorsal	Anterior	Posterior	Ventral	1	2
03-417	15.11		■		■						■	■
03-1778	15.32	■	■	■	■							
03-2015	15.59				■							
03-890	15.67		■		■							
03-1515	15.98		■		■							
03-372	16.2	■	■	■	■							
03-1438	16.42		■		■						■	■
03-2684	16.43	■	■		■						■	■
03-1480	17.15				■		■					
03-398	17.2				■							
03-1204	17.22		■		■							
03-2756	17.47		■	■	■		■					
03-210	17.55	■	■		■						■	
03-1976	17.55	■		■	■							
03-319	17.58		■		■						■	■
03-1897	17.7		■		■							
03-1408	17.9		■		■							
03-1252	18.1	■	■	■	■							
03-435	18.46				■		■				■	■
03-654	18.55		■		■							
03-2033	18.86		■		■						■	■
03-2001	18.91	■	■	■	■						■	
03-2015	19.2			■	■							
03-503	19.34				■						■	■

Continued on next page

Continued from previous page

Specimen MHNM	Estimated TL (mm)	Otic plates	Vertebral structures	Scapulo-coracoid	Body scales	Nasal bones	Sclerotic bones				Branchiostegal rays	
							Dorsal	Anterior	Posterior	Ventral	1	2
03-1427	19.5		■	■	■						■	■
03-1916	19.58				■							
03-2599	19.93				■						■	■
03-1516	20.12		■		■							
03-401	20.14		■		■						■	■
03-354	20.3				■							
03-1916	20.35		■	■	■							
03-1881	20.59	■		■	■							
03-1378	20.65		■	■	■							
03-1250	21.37		■	■	■						■	■
03-433	21.55		■	■	■							
03-2684	21.57	■		■	■						■	■
03-549	21.64		■		■		■				■	■
03-978	21.64	■	■		■							
03-740	21.92			■	■							
03-531	22.43		■	■	■		■				■	■
03-1961	22.48		■	■	■							
03-561	22.59				■							
03-1963	22.73			■	■							
03-1192	22.75		■		■							
03-2570	22.96		■	■	■						■	
03-1515	23.15				■						■	■
03-1205	23.47	■	■	■	■	■						
03-1833	23.75				■							
03-1962	23.79			■	■							
03-363	23.88		■	■	■	■	■					

Continued on next page

Continued from previous page

Specimen MHNM	Estimated TL (mm)	Otic plates	Vertebral structures	Scapulo-coracoid	Body scales	Nasal bones	Sclerotic bones				Branchiostegal rays	
							Dorsal	Anterior	Posterior	Ventral	1	2
03-1819	23.9		■	■	■						■	■
03-813	23.9	■	■		■							
03-2480	24.05			■	■							
03-1236	24.25	■	■		■						■	■
03-432	24.31		■	■	■							
03-2477	24.55		■	■	■							
03-916	24.7			■	■		■	■			■	■
03-2483	24.86		■	■	■							
03-1985	24.93			■	■							
03-1509	24.95				■	■	■				■	■
03-2440	25.12			■	■							
03-450	25.21		■		■							
03-558	25.3			■	■		■				■	■
03-1879	25.3		■		■						■	■
03-2629	25.51			■	■						■	■
03-1847	25.53	■	■		■							
03-25	25.57			■	■						■	■
03-1100	25.88			■	■	■	■				■	■
03-1311	25.97			■	■							
03-2660	25.97			■	■							
03-220	26.16			■	■	■					■	■
03-2025	26.27		■	■	■							
03-1538	26.28		■	■	■							
03-78	26.52				■						■	■
03-235	26.69			■	■		■	■	■		■	
03-2654	26.73	■	■		■							

Continued on next page

Continued from previous page

Specimen MHNM	Estimated TL (mm)	Otic plates	Vertebral structures	Scapulo-coracoid	Body scales	Nasal bones	Sclerotic bones				Branchiostegal rays	
							Dorsal	Anterior	Posterior	Ventral	1	2
03-695	26.87											
03-108	27.58											
03-1406	27.79											
03-2030	27.84											
03-2487	27.88											
03-369	27.96											
03-773	28.13											
03-1295	28.2											
03-1496	28.2											
03-2589	28.36											
03-2613	28.62											
03-1969	28.73											
03-1306	28.79											
03-123	29.13											
03-176	29.2											
03-2630	29.29											
03-867	29.34											
03-699	29.36											
03-1994	29.37											
03-164	29.47											
03-1051	29.49											
03-2025	29.57											
03-2612	29.63											
03-551	29.71											
03-1909	29.72											
03-336	29.87											

Continued on next page

Continued from previous page

Specimen MHNM	Estimated TL (mm)	Otic plates	Vertebral structures	Scapulo-coracoid	Body scales	Nasal bones	Sclerotic bones				Branchiostegal rays	
							Dorsal	Anterior	Posterior	Ventral	1	2
03-1687	30.24			■	■		■	■	■			
03-217	31.12			■	■	■	■	■			■	■
03-868	31.17			■	■	■	■	■	■	■	■	■
03-716	31.34			■	■	■	■	■	■		■	■
03-2631	31.47		■	■	■							
03-1008	31.53		■	■	■							
03-1314	31.64		■	■	■						■	
03-55	31.71			■	■	■	■				■	■
03-2589	32.42			■	■						■	■
03-1550	33.1			■	■	■						
03-701	33.18		■	■	■							
03-1533	33.29		■		■							
03-38	33.64			■	■	■	■				■	■
03-530	33.7			■	■		■	■	■		■	■
03-2614	33.74			■	■		■				■	■
03-1798	34.34			■	■							
03-723	34.48			■	■	■	■	■			■	■
03-88	35.12			■	■	■	■	■	■	■	■	■
03-2620	35.44			■	■	■					■	■
03-597	35.6			■	■	■	■	■	■		■	■
03-570	35.75				■							
03-1083	35.92				■							
03-98	35.93			■	■	■	■				■	■
03-452	36.19			■	■		■	■			■	■
03-1233	36.29			■	■							
03-1215	36.35			■	■						■	■

Continued on next page

Continued from previous page

Specimen MHNM	Estimated TL (mm)	Otic plates	Vertebral structures	Scapulo-coracoid	Body scales	Nasal bones	Sclerotic bones				Branchiostegal rays	
							Dorsal	Anterior	Posterior	Ventral	1	2
03-1031	36.4											
03-933	36.41											
03-1731	37.11											
03-388	37.6											
03-1545	38.21											
03-525	38.3											
03-2719	38.41											
03-971	39.27											
03-584	39.31											
03-2621	40.8											
03-534	40.98											
03-347	41.2											
03-2637A	41.2											
03-2761	41.29											
03-198	42.5											
03-529	43.28											
03-528	43.6											
03-139	43.8											
03-58	44											
03-1497	45.29											
03-715	45.8											
03-961	46.45											
03-1817	47.51											
03-1971	49.21											
03-2679	49.27											
03-1107	52.72											

Continued on next page

Continued from previous page

Specimen MHNM	Estimated TL (mm)	Branchiostegal rays										Supra-orbital sc	Sub-orbital sc
		3	4	5	6	7	8	9	10	11	12		
03-94	4.5												
03-403	4.51												
01-260	5.5												
01-250	6.64												
03-843	6.79												
03-440	6.9												
03-1292	7.17												
03-440	7.29												
01-271	7.82												
03-403	8.12												
03-440	8.76												
01-275	9.14												
03-2729	9.94												
01-256	9.99												
03-1292	10.84												
01-253	11.4												
03-2684	12.71												
03-316	13.58												
01-230	13.6												
03-259	13.82												
03-622	14.1												
03-418	14.17												
03-1378	14.65												
01-230	15.1												

Continued on next page

Continued from previous page

Specimen MHNM	Estimated TL (mm)	Branchiostegal rays										Supra-orbital sc	Sub-orbital sc
		3	4	5	6	7	8	9	10	11	12		
03-417	15.11												
03-1778	15.32												
03-2015	15.59												
03-890	15.67												
03-1515	15.98												
03-372	16.2												
03-1438	16.42												
03-2684	16.43												
03-1480	17.15												
03-398	17.2												
03-1204	17.22												
03-2756	17.47												
03-210	17.55												
03-1976	17.55												
03-319	17.58												
03-1897	17.7												
03-1408	17.9												
03-1252	18.1												
03-435	18.46												
03-654	18.55												
03-2033	18.86												
03-2001	18.91												
03-2015	19.2												
03-503	19.34												

Continued on next page

Continued from previous page

Specimen MHNM	Estimated TL (mm)	Branchiostegal rays										Supra-orbital sc	Sub-orbital sc
		3	4	5	6	7	8	9	10	11	12		
03-1427	19.5												
03-1916	19.58												
03-2599	19.93												
03-1516	20.12												
03-401	20.14												
03-354	20.3												
03-1916	20.35												
03-1881	20.59												
03-1378	20.65												
03-1250	21.37												
03-433	21.55												
03-2684	21.57												
03-549	21.64												
03-978	21.64												
03-740	21.92												
03-531	22.43												
03-1961	22.48												
03-561	22.59												
03-1963	22.73												
03-1192	22.75												
03-2570	22.96												
03-1515	23.15												
03-1205	23.47												
03-1833	23.75												
03-1962	23.79												
03-363	23.88												

Continued on next page

Continued from previous page

Specimen MHNM	Estimated TL (mm)	Branchiostegal rays										Supra-orbital sc	Sub-orbital sc
		3	4	5	6	7	8	9	10	11	12		
03-1819	23.9												
03-813	23.9												
03-2480	24.05												
03-1236	24.25												
03-432	24.31												
03-2477	24.55												
03-916	24.7												
03-2483	24.86												
03-1985	24.93												
03-1509	24.95												
03-2440	25.12												
03-450	25.21												
03-558	25.3												
03-1879	25.3												
03-2629	25.51												
03-1847	25.53												
03-25	25.57												
03-1100	25.88												
03-1311	25.97												
03-2660	25.97												
03-220	26.16												
03-2025	26.27												
03-1538	26.28												
03-78	26.52												
03-235	26.69												
03-2654	26.73												

Continued on next page

Continued from previous page

Specimen MHNM	Estimated TL (mm)	Branchiostegal rays										Supra-orbital sc	Sub-orbital sc
		3	4	5	6	7	8	9	10	11	12		
03-695	26.87												
03-108	27.58												
03-1406	27.79												
03-2030	27.84												
03-2487	27.88												
03-369	27.96												
03-773	28.13												
03-1295	28.2												
03-1496	28.2												
03-2589	28.36												
03-2613	28.62												
03-1969	28.73												
03-1306	28.79												
03-123	29.13												
03-176	29.2												
03-2630	29.29												
03-867	29.34												
03-699	29.36												
03-1994	29.37												
03-164	29.47												
03-1051	29.49												
03-2025	29.57												
03-2612	29.63												
03-551	29.71												
03-1909	29.72												
03-336	29.87												

Continued on next page

Continued from previous page

Specimen MHNM	Estimated TL (mm)	Branchiostegal rays										Supra-orbital sc	Sub-orbital sc
		3	4	5	6	7	8	9	10	11	12		
03-1687	30.24	■	■	■	■								
03-217	31.12											■	
03-868	31.17	■	■	■								■	
03-716	31.34	■	■	■	■	■	■	■	■	■	■		
03-2631	31.47	■	■	■	■	■	■	■	■				
03-1008	31.53												
03-1314	31.64												
03-55	31.71											■	
03-2589	32.42	■											
03-1550	33.1	■	■	■	■	■	■						
03-701	33.18												
03-1533	33.29												
03-38	33.64											■	
03-530	33.7	■	■	■	■	■	■	■					
03-2614	33.74	■	■									■	
03-1798	34.34	■	■	■									
03-723	34.48											■	
03-88	35.12	■	■	■	■	■	■					■	
03-2620	35.44	■											
03-597	35.6	■	■	■	■	■						■	
03-570	35.75	■	■	■	■	■	■	■	■	■			
03-1083	35.92												
03-98	35.93											■	
03-452	36.19	■	■	■	■								
03-1233	36.29	■	■	■	■	■	■						
03-1215	36.35											■	

Continued on next page

Continued from previous page

Specimen MHNM	Estimated TL (mm)	Branchiostegal rays										Supra-orbital sc	Sub-orbital sc
		3	4	5	6	7	8	9	10	11	12		
03-1031	36.4												
03-933	36.41												
03-1731	37.11												
03-388	37.6												
03-1545	38.21												
03-525	38.3												
03-2719	38.41												
03-971	39.27												
03-584	39.31												
03-2621	40.8												
03-534	40.98												
03-347	41.2												
03-2637A	41.2												
03-2761	41.29												
03-198	42.5												
03-529	43.28												
03-528	43.6												
03-139	43.8												
03-58	44												
03-1497	45.29												
03-715	45.8												
03-961	46.45												
03-1817	47.51												
03-1971	49.21												
03-2679	49.27												
03-1107	52.72												

Continued on next page

Continued from previous page

Specimen MHNM	Estimated TL (mm)	Profundus scales	Otic scales	Otic comm. sc	Pectoral spines	Interm. spines	Pelvic spines	Pelvic scales	Anal spine	Anal scales	Dorsal spine	Dorsal scales	Hypoch. scales
03-94	4.5												
03-403	4.51												
01-260	5.5												
01-250	6.64												
03-843	6.79												
03-440	6.9												
03-1292	7.17												
03-440	7.29												
01-271	7.82												
03-403	8.12												
03-440	8.76												
01-275	9.14												
03-2729	9.94												
01-256	9.99												
03-1292	10.84												
01-253	11.4												
03-2684	12.71												
03-316	13.58												
01-230	13.6												
03-259	13.82												
03-622	14.1												
03-418	14.17												
03-1378	14.65												
01-230	15.1												

Continued on next page

Continued from previous page

Specimen MHNM	Estimated TL (mm)	Profundus scales	Otic scales	Otic comm. sc	Pectoral spines	Interm. spines	Pelvic spines	Pelvic scales	Anal spine	Anal scales	Dorsal spine	Dorsal scales	Hypoch. scales
03-417	15.11												
03-1778	15.32												
03-2015	15.59												
03-890	15.67												
03-1515	15.98												
03-372	16.2												
03-1438	16.42												
03-2684	16.43												
03-1480	17.15												
03-398	17.2												
03-1204	17.22												
03-2756	17.47												
03-210	17.55												
03-1976	17.55												
03-319	17.58												
03-1897	17.7												
03-1408	17.9												
03-1252	18.1												
03-435	18.46												
03-654	18.55												
03-2033	18.86												
03-2001	18.91												
03-2015	19.2												
03-503	19.34												

Continued on next page

Continued from previous page

Specimen MHNM	Estimated TL (mm)	Profundus scales	Otic scales	Otic comm. sc	Pectoral spines	Interm. spines	Pelvic spines	Pelvic scales	Anal spine	Anal scales	Dorsal spine	Dorsal scales	Hypoch. scales
03-1427	19.5												
03-1916	19.58												
03-2599	19.93												
03-1516	20.12												
03-401	20.14												
03-354	20.3												
03-1916	20.35												
03-1881	20.59												
03-1378	20.65												
03-1250	21.37												
03-433	21.55												
03-2684	21.57												
03-549	21.64												
03-978	21.64												
03-740	21.92												
03-531	22.43												
03-1961	22.48												
03-561	22.59												
03-1963	22.73												
03-1192	22.75												
03-2570	22.96												
03-1515	23.15												
03-1205	23.47												
03-1833	23.75												
03-1962	23.79												
03-363	23.88												

Continued on next page

Continued from previous page

Specimen MHNM	Estimated TL (mm)	Profundus scales	Otic scales	Otic comm. sc	Pectoral spines	Interm. spines	Pelvic spines	Pelvic scales	Anal spine	Anal scales	Dorsal spine	Dorsal scales	Hypoch. scales
03-1819	23.9												
03-813	23.9												
03-2480	24.05												
03-1236	24.25												
03-432	24.31												
03-2477	24.55												
03-916	24.7												
03-2483	24.86												
03-1985	24.93												
03-1509	24.95												
03-2440	25.12												
03-450	25.21												
03-558	25.3												
03-1879	25.3												
03-2629	25.51												
03-1847	25.53												
03-25	25.57												
03-1100	25.88												
03-1311	25.97												
03-2660	25.97												
03-220	26.16												
03-2025	26.27												
03-1538	26.28												
03-78	26.52												
03-235	26.69												
03-2654	26.73												

Continued on next page

Continued from previous page

Specimen MHNM	Estimated TL (mm)	Profundus scales	Otic scales	Otic comm. sc	Pectoral spines	Interm. spines	Pelvic spines	Pelvic scales	Anal spine	Anal scales	Dorsal spine	Dorsal scales	Hypoch. scales
03-695	26.87												
03-108	27.58												
03-1406	27.79												
03-2030	27.84												
03-2487	27.88												
03-369	27.96												
03-773	28.13												
03-1295	28.2												
03-1496	28.2												
03-2589	28.36												
03-2613	28.62												
03-1969	28.73												
03-1306	28.79												
03-123	29.13												
03-176	29.2												
03-2630	29.29												
03-867	29.34												
03-699	29.36												
03-1994	29.37												
03-164	29.47												
03-1051	29.49												
03-2025	29.57												
03-2612	29.63												
03-551	29.71												
03-1909	29.72												
03-336	29.87												

Continued on next page

Continued from previous page

n MHNM	Estimated TL (mm)	Profundus scales	Otic scales	Otic comm. sc	Pectoral spines	Interm. spines	Pelvic spines	Pelvic scales	Anal spine	Anal scales	Dorsal spine	Dorsal scales	Hypoch. scales
03-1687	30.24												
03-217	31.12												
03-868	31.17												
03-716	31.34												
03-2631	31.47												
03-1008	31.53												
03-1314	31.64												
03-55	31.71												
03-2589	32.42												
03-1550	33.1												
03-701	33.18												
03-1533	33.29												
03-38	33.64												
03-530	33.7												
03-2614	33.74												
03-1798	34.34												
03-723	34.48												
03-88	35.12												
03-2620	35.44												
03-597	35.6												
03-570	35.75												
03-1083	35.92												
03-98	35.93												
03-452	36.19												
03-1233	36.29												
03-1215	36.35												

Continued on next page

Continued from previous page

Specimen MHNM	Estimated TL (mm)	Profundus scales	Otic scales	Otic comm. sc	Pectoral spines	Interm. spines	Pelvic spines	Pelvic scales	Anal spine	Anal scales	Dorsal spine	Dorsal scales	Hypoch. scales
03-1031	36.4				■		■	■	■	■	■	■	■
03-933	36.41				■		■		■	■	■	■	
03-1731	37.11				■	■	■		■		■		
03-388	37.6				■		■				■	■	
03-1545	38.21				■	■	■		■		■		■
03-525	38.3				■		■	■	■	■	■		■
03-2719	38.41				■	■	■				■		■
03-971	39.27				■		■	■	■	■	■	■	
03-584	39.31	■			■		■		■			■	
03-2621	40.8				■		■		■	■	■	■	
03-534	40.98				■	■	■		■	■	■		■
03-347	41.2		■	■	■		■		■			■	
03-2637A	41.2				■	■	■		■		■		■
03-2761	41.29				■		■	■	■	■	■	■	■
03-198	42.5				■	■	■		■		■		■
03-529	43.28				■		■		■				■
03-528	43.6				■		■		■		■	■	■
03-139	43.8				■	■	■		■		■		
03-58	44				■				■		■		■
03-1497	45.29				■		■		■		■	■	■
03-715	45.8		■		■		■		■		■	■	■
03-961	46.45				■		■		■	■	■		
03-1817	47.51				■		■		■				■
03-1971	49.21				■		■		■		■	■	■
03-2679	49.27		■		■							■	
03-1107	52.72				■		■				■	■	

Continued on next page

Continued from previous page

	Eye lenses	Semi-circular canals	Otoliths			Nasal plates	Palatoquadrate	Meckel's cartilage
			Saccular	Lagenar	Utricular			
Observed	157	119	133	93	41	51	12	8
Expected	178	172	177	177	176	176	110	88
RE (%)	**88.2**	**69.19**	**75.14**	**52.54**	23.30	28.98	10.1	9.09
Max. Gap	3	7	5	7	19	24	26	36
Position	end	early	late	middle	end	middle	middle	middle
TL 1st appearance (mm)	4.5	7.17	4.51	4.51	5.5	5.5	22.96	25.57

	Otic plates	Vertebral structures	Scapulo-coracoid	Body scales	Nasal bones	Sclerotic bones				Branchiostegal rays	
						Dorsal	Anterior	Posterior	Ventral	1	2
Observed	2	63	112	162	36	50	26	13	4	85	77
Expected	18	178	168	162	108	146	98	80	50	178	178
RE (%)	11.11	35.39	**66.67**	**100**	33.33	34.25	26.53	16.25	8	47.75	43.26
Max. Gap	11	40	6	NA	9	17	17	9	27	10	16
Position	late	late	early	NA	early	early	early	early	late	early	early
TL 1st appearance (mm)	39.31	4.5	8.76	12.71	23.47	17.15	24.7	26.69	31.17	4.5	4.5

Continued on next page

Continued from previous page

	Branchiostegal rays										Supra-orbital sc	Infra-orbital sc
	3	4	5	6	7	8	9	10	11	12		
Observed	63	49	45	36	32	23	15	13	11	7	29	2
Expected	157	148	140	136	136	95	95	95	95	95	87	18
RE (%)	40.13	33.11	32.14	26.47	23.53	24.21	15.79	13.68	11.58	7.37	33.33	11.11
Max. Gap	10	22	22	22	22	11	11	15	16	47	10	11
Position	early	middle	middle	early	early	late	late	middle	middle	end	middle	late
TL 1st appearance (mm)	14.17	16.42	17.58	18.46	18.46	24.95	24.95	24.95	24.95	24.95	25.88	39.31

	Profundus scales	Otic scales	Otic comm. scales	Pectoral spines	Interm. spines	Pelvic spines	Pelvic scales	Anal spine	Anal scales	Dorsal spine	Dorsal scales	Hypoch. scales
Observed	1	5	2	148	35	127	13	133	23	123	34	88
Expected	18	55	55	174	116	161	88	173	88	174	159	158
RE (%)	5.56	9.09	3.64	**85.06**	30.17	**78.88**	14.77	**76.88**	26.14	**70.69**	21.38	**55.7**
Max. Gap	NA	33	39	5	18	6	16	4	12	7	19	6
Position	NA	middle	middle	early	middle	early	middle	early	middle	early	early	early
TL 1st appearance (mm)	39.31	29.71	29.71	6.79	21.92	13.58	25.57	6.9	25.57	6.79	13.82	14.1

Text S1. Reliability Index

Inter-individual variation in developmental sequence (i.e., timing and order of appearance of events) has been reported in developmental sequences of living organisms (Colbert and Rowe 2008; Maxwell 2008; de Jong et al. 2009; Fischer-Rousseau *et al.* 2009). Here, we developed a reliability estimate (RE) which is calculated for each developmental event rather than for the complete sequence because non-developmental sources of variation have the potential to be included in fossilized ontogenies (e.g., taphonomic alteration). Departure from the expected occurrence of skeletal events within a growth series owing to inter-individual growth difference has been designate as disparity by dividing the total number of non-occurrences of events after their initial appearance by the total number of occurrences for all events (Maisano 2002). The RE is calculated for each structure by dividing the actual number of specimens having an anatomical structure by the number of specimens expected to have this structure [i.e., number of specimens longer (in terms of TL) than the smallest specimen that displays the structure] (Table S1). Four parameters are calculated for each anatomical structure forming a sequence: (1) the actual number of specimens in which a structure has been observed; (2) the expected number of specimens in which the structure should be present; and the maximal gap in the sequence in terms of (3) the number of specimens lacking the structure, and (4) the difference in TL (i.e., the difference in TL between the specimens at each extremities of the gap) (Table S1). The maximal gap is also positioned relatively to the sequence (i.e., early, middle, late). Only the specimens for which the parts are preserved are taken into account in these four parameters. A high RE (> 50%) means that many specimens expected to have a structure do have it and that the variation for this structure is low. A low RE (< 50%) suggests the sample might be less reliable for this structure, especially when there is an important gap in the early part of the ontogenetic sequence.

Table S2. Raw data (in percentage of weight) from EDS X-ray analyses in *Triazeugacanthus affinis*. Data for each skeletal structures is recorded from the mean of several punctual spectra. Traces include elements which are not implied in the biomineral but are present in the SEM chamber as the EDS Xray is an environmental one or are remains of sedimentary matrix.

		C	O	Ca	P	F	Traces	# of spectra
**MHNM 03-440 #2**	**Head structures**	59.46	34.93				5.71	2
	**Otoliths**	15.98	61.72	13.73			8.57	2
	**Scapula**	16.22	44.9	19.02	8.72	2.85	8.29	5
	**Pectoral spine**	16.98	43.37	19.47	9.88	5.54	4.76	2
	**Vertebral structures**	59.23	29.24	5.17			6.36	1
**MHNM 03-440 #1**	**Head structures**	66.49	21.34	1.12			1.05	2
	**Otoliths**	15.95	56.25	23.65			4.15	2
**MHNM 03-398 scales**	**Endoskeleton**	21.66	16.98	56.41			4.95	1
	**Scale inner layer**	36.06	20.15	34.03	5.72		4.04	2
	**Scale outer layer**	42.04	19.89	32.03	2.63		3.41	1
	**Scale surface**	33.76	21	38.11	3.51		3.62	1
**MHNM 03-398 anal spine**	**Vascular cavity**	32.59	18.89	45.50			1.02	1
	**Endoskeleton**	31.63	16.84	48.65			2.88	2
	**Spine outer layer**	40.03	13.06	39.45	1.29		6.17	1
	**Spine piece**	39.14	15.77	39.45	3.11		2.53	1
	**Spine inner layer**	36.70	17.05	38.01	3.09		5.15	3
**MHNM 03-1497**	**Sclerotic bone**	22.02	41.72	20.40	9.89	3.67	2.3	1
	**Palatoquadrate**	25.82	42.08	16.40	9.26	6.17	0.27	2
	**Branchiostegal rays**	27.42	43.11	13.72	7.54	6.32	1.89	2
	**Scapula**	11.10	50.19	20.74	10.02	6.26	1.69	3
	**Pectoral spine**	28.68	45.25	12.65	6.77	4.68	1.97	3
	**Scales**	13.10	47.99	20.03	8.75	7.95	2.18	4

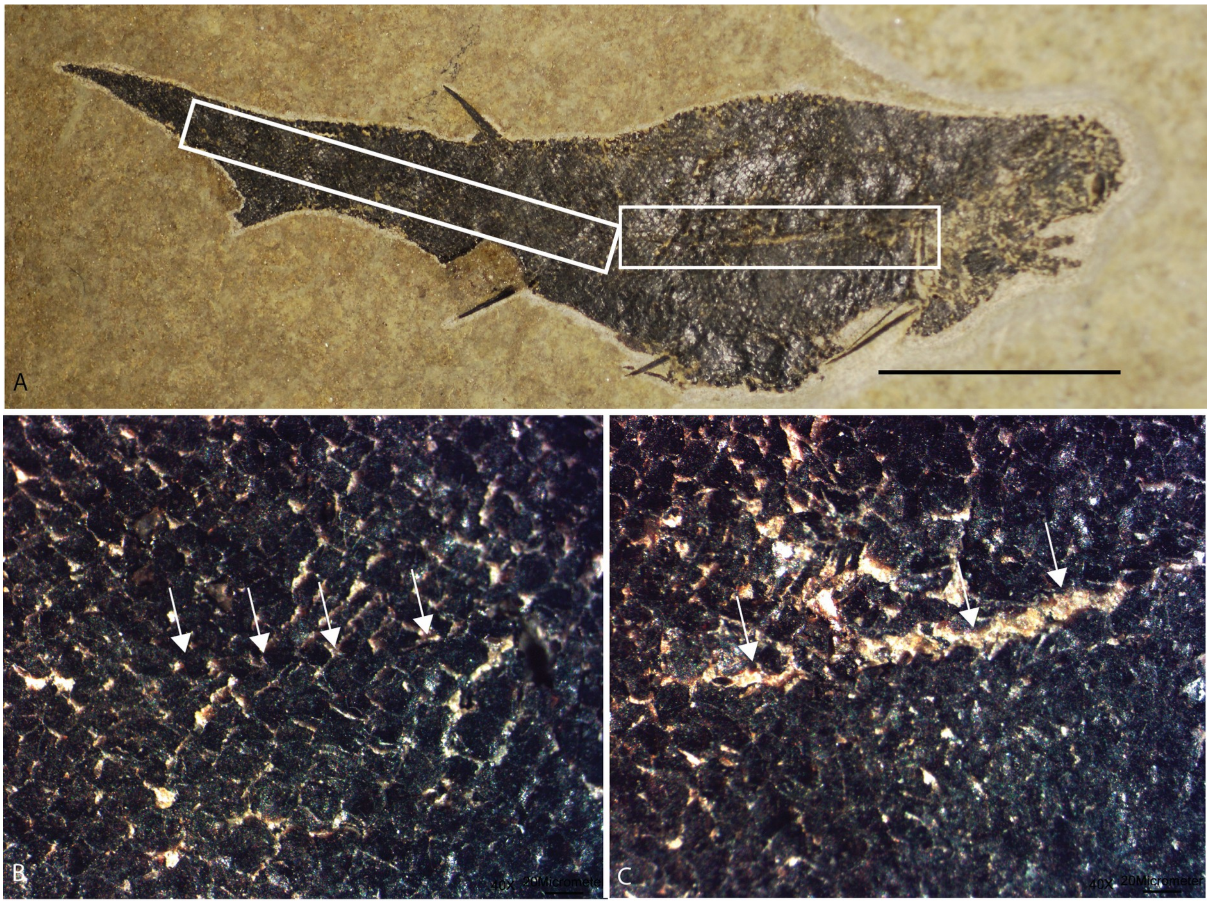

Figure S2. ***Triazeugacanthus* lateral line canal and scales.** MHNM 03-1497 A. Anterior and posterior portion of the lateral line canal, morphologically different concerning the arrangement of scales. Anterior rectangle: gap between the rows of scales surrounding the lateral line. Posterior rectangle: no such a gap. B. Close-up of the posterior region. C. Close-up of the anterior region. Arrows indicate the position of the lateral line canal. Scale bar = 10 mm. Photo: Marion Chevrinais.

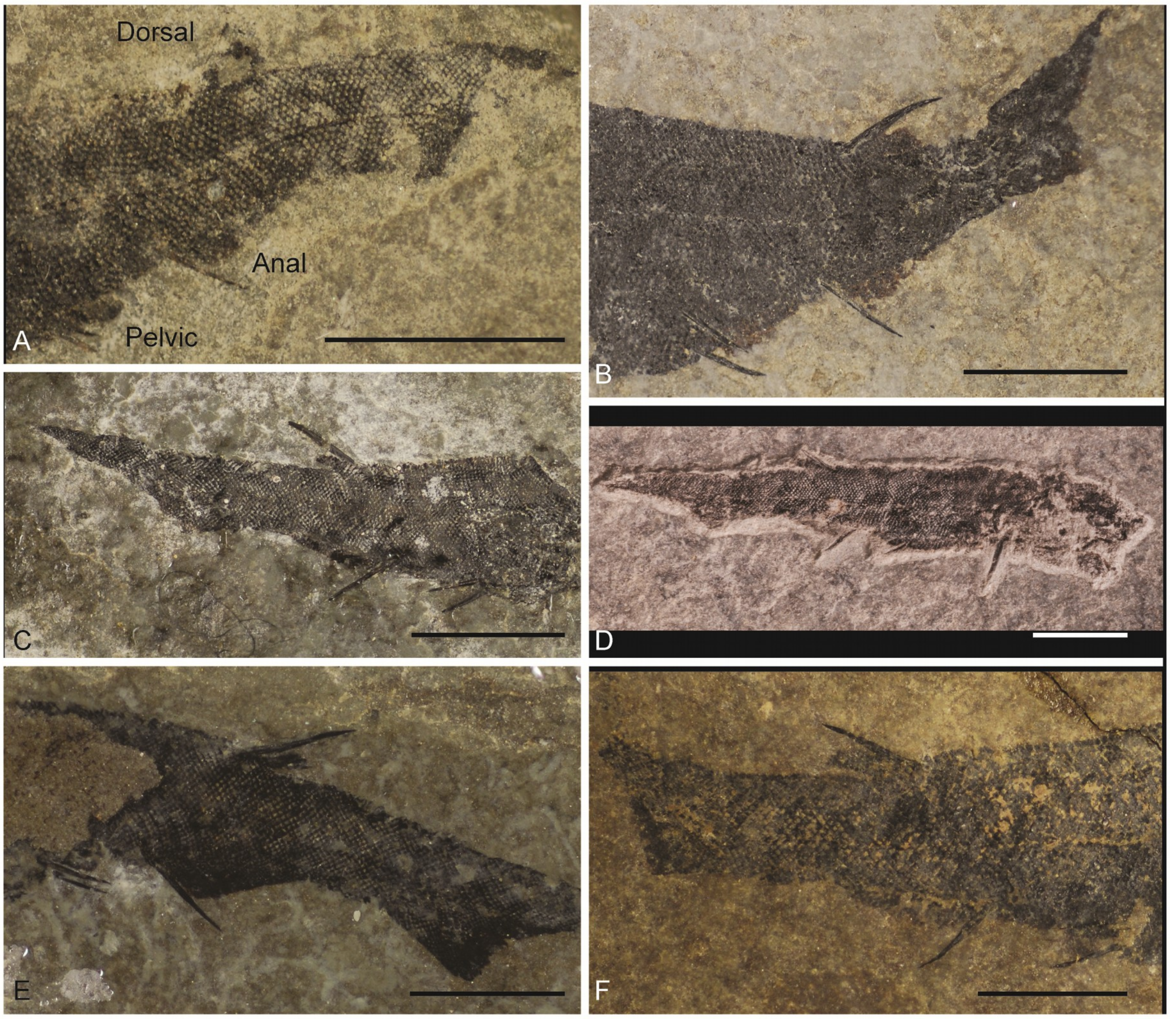

Figure S3. Pelvic, anal and dorsal (A) fin webs in *Triazeugacanthus*. (A) MHNM 03-25. (B) MHNM 03-867. (C) MHNM 03-220. (D) MHNM 03-2589 2. (E) MHNM 03-525. (F) MHNM 03-2761. Scale bars = 5 mm. Photo: Marion Chevrinais.

Table S3. Elongation ratio in various chondrichthyan species.

Length and depth of chondrichthyan species collected from Wyffels (2009).

Species	Stage	Length (mm)	Depth (mm)	Elongation ratio
Squalus acanthias	Embryo	120	13.8	8.7
	Juvenile	230	24.3	9.5
	Adult	1010	110	9.1
Lamna nasus	Embryo	394	59	6.7
	Juvenile	580	125	4.6
	Adult	840	190	4.4
Carcharias taurus	Embryo	131	17	7.7
	Juvenile	271	43	6.3
	Adult	1110	170	6.5
Callorhinchus milii	Embryo	75	7.1	10.7
	Embryo	105	12	8.7
	Juvenile	133	24	5.5
	Adult	920	137	6.7

Table S4. Raw data for Reliability index calculation in *Lodeacanthus gaujicus* Upeniece (2011)

Specimen LDM	TL (mm)	Scapula	Endocranium	Jaws	Branch arches	Branch rays	Circumb bones	Nasals	Cheek bones	Tesserae	SL trunk	SL head
270/45	~8											
270/28	~13.6											
270/25	~19.6											
270/22	~20.5											
270/23	~20.6											
270/3	~20.8											
270/12	23											
270/37	25											
270/2	NA											
270/19	34.7											
270/38	37.6											
270/41	38.3											
270/9	38.9											
270/14	NA											
Observed		13	10	7	2	14	11	7	9	7	7	5
Expected		14	13	14	6	14	13	13	13	8	12	11
RE (%)		**92.86**	**76.92**	**50**	33.33	100	**84.62**	**53.85**	**69.23**	**87.5**	**58.33**	45.45
Max. Gap		1	1	0	4	0	1	2	2	1	3	6
Position		early	early		late			early and late	middle	late	late	late
TL 1st appearance (mm)		8	13.6	25	?	8	13.6	13.6	13.6	23	19.6	20.5

References

COLBERT, M. W. and ROWE, T. 2008. Ontogenetic sequence analysis: Using parsimony to characterize developmental sequences and sequence polymorphism. *Journal of Experimental Zoology Part B: Molecular and Developmental Evolution*, **310B**, 398–416.

FISCHER-ROUSSEAU, L., CLOUTIER, R. and ZELDITCH, M. L. 2009. Morphological integration and developmental progress during fish ontogeny in two contrasting habitats. *Evolution & Development*, **11**, 740–753.

DE JONG, I. M. L., COLBERT, M. W., WITTE, F. and RICHARDSON, M. K. 2009. Polymorphism in developmental timing: Intraspecific heterochrony in a Lake Victoria cichlid. *Evolution & Development*, **11**, 625–635.

MAISANO, J. 2002. The potential utility of postnatal skeletal developmental patterns in squamate phylogenetics. *Zoological Journal of the Linnean Society*, **136**, 277–313.

MAXWELL, E. E. 2008. Ossification sequence of the avian order Anseriformes, with comparison to other precocial birds. *Journal of Morphology*, **269**, 1095–1113.

UPENIECE, I. 2011. Palaeoecology and juvenile individuals of the Devonian placoderm and acanthodian fishes from Lode site, Latvia.Latvia, University of Latviapp.

WYFFELS, J. T. 2009. Embryonic development of chondrichthyan fishes—a review. *Development of non-Teleost fishes. Enfield: Science Publishers*, 1–103.
